# Modulating Thermal Stability and Flexibility in Chitosan Films with Neutral Polyol-Boric Acid Complexes

**DOI:** 10.1021/acs.biomac.5c00177

**Published:** 2025-06-26

**Authors:** Olivia E. Coer, Brandy L. Davidson, Brycelyn M. Boardman, Gretchen M. Peters

**Affiliations:** Department of Chemistry and Biochemistry, 3745 James Madison University, Harrisonburg, Virginia 22807, United States

## Abstract

The incorporation of boron into bioplastics offers the potential for diverse applications, with the structure–property relationship between polymer chains and boron species being the key for design. Here, we report the ability to modulate the flexibility and thermal stability of chitosan materials by varying the concentrations of erythritol and the molar equivalents of boric acid. Erythritol and boric acid form neutral complexes that alter the hydrogen-bonding face of erythritol while maintaining free diol units. 1D and 2D NMR experiments indicate preferential formation of the 1,3-isomer (85%) with minor amounts of 1,2- and 2,3-isomers. Structural, thermal, mechanical, and morphological characterization was performed using ATR-FTIR, TGA and DSC, DMA, and SEM, respectively. Molecular-level interactions of the complexes and d-glucosamine, the repeat unit of chitosan, showed increased aggregation and hydrogen-bonding interactions of the free diol units with the NH of d-glucosamine, supporting the trends in flexibility observed in the polymer system.

## Graphical abstract

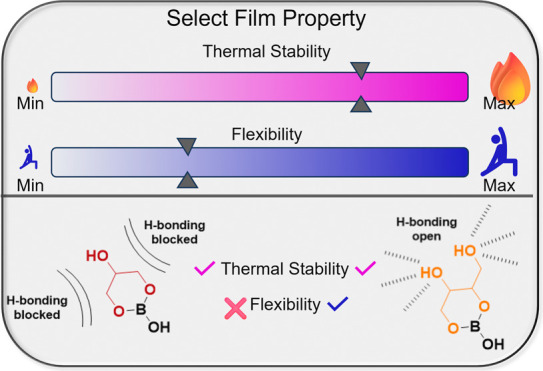

## Introduction

Boron-containing compounds, such as boronic acids and boric acid (BA), have been effective in sensing and drug delivery applications.^1^ The ability to form B–O esters with the 1,2- or 1,3-diols present in substrates including polysaccharides, glycoproteins, glycated proteins, and dopamine make them promising for sensing and diagnostic medicine.^2,3^ Furthermore, the reversible nature of the B–O ester bond provides a mechanism for controlled release in drug delivery^4,5^ and has also been shown to be an effective and labile cross-linker in self-healing materials^6–9^ and hydrogels.^10–15^ Incorporation of boronic acid groups into hydrogels and copolymers has also been shown to change the properties of the materials by increasing the hydrophilicity.^16,17^ From these studies, it is obvious that the incorporation of boron-containing compounds into materials results in novel properties and expanded function. Thus, developing an in-depth understanding of the structure–property relationship of such materials remains key for application optimization and implementation.^18^

Chitosan is a promising biopolymer with a wide breadth of potential applications.^19–21^ Its biocompatible nature and antibacterial properties allow for chitosan to be utilized in food packaging^22^ and sensing as well as pharmaceutical^23,24^ and orthopedic applications.^25–27^ The use of boric or boronic acids has also been explored as cross-linkers for chitosan blend materials^28–31^ and as a means of expanding the function of chitosan for sensing applications.^32^ Chitosan functionalized with phenylboronic acid has been effective for tumor targeting and delivery of cancer therapies.^33,34^ Furthermore, the incorporation of boric acid into chitosan materials has been shown to increase flame retardancy and introduce antibacterial and wrinkle-free properties in fabrics.^35^ In chitosan-based bioplastics, small molecule plasticizers, such as glycerol (Glyc), are added to plasticize and improve the material properties by disrupting the strong interchain polymer–polymer hydrogen-bonding interactions. When plasticized, chitosan films typically display an increase in flexibility and ductility with a decrease in the crystallinity, storage modulus, and thermal transition temperatures.^36–38^ Recently, we investigated the addition of boric acid (BA) into Glyc-plasticized chitosan. Since the formation of complexes between polyols and BA has been well-established, we anticipated that the complexion of Glyc with BA would alter the binding face of the plasticizer and thus the properties of the material would be impacted.^39^ Indeed, when BA was introduced to Glyc-containing chitosan films, the resulting materials showed a significant increase in stiffness, higher decomposition temperatures, and water solubility when compared to materials with Glyc alone. We also observed that the addition of BA to these systems had a significant impact on the flexibility of films, consistent with a change in the hydrogen-bonding capacity of the plasticizer. Our previous molecular-level studies with the repeat unit of chitosan, d-glucosamine (GlcN), indicated that specific hydrogen-bonding interactions are critical for effective plasticization.^40^ Additionally, we found that variations in the diol-GlcN interactions can dictate the mechanism of plasticization and ultimately the flexibility of the material. These studies highlighted the differences in interactions with the NH and OH functionalities of GlcN based on primary diol binding mode suggesting that 1,3-diol binding prefers NH interactions where 1,2-diol binding prefers OH interactions.^41^ These combined studies suggest that the lack of a diol binding unit in the Glyc-BA complex impedes the hydrogen-bonding interactions needed for effective plasticization.

Herein, we describe the modulation of the flexibility and thermal stability of chitosan materials using erythritol (Ery) and BA. Ery was chosen for this study because it is structurally similar to Glyc and can form neutral boron complexes in the presence of BA. However, because Ery is a tetrol rather than a triol, we theorized that formation of neutral boron complexes with Ery would result in the retention of free diol binding units, which would be capable of interactions previously “turned off” in Glyc-BA systems (Figure 1). We utilized advanced NMR studies to elucidate the structures of neutral boron complexes formed with Ery and BA, all of which indeed have free diol units. The resulting chitosan materials with Ery-BA complexes display increased flexibility and thermal stability relative to Glyc-BA materials and can be tuned based on Ery concentration with varying BA equivalents. Molecular-level studies between GlcN and Ery-BA indicate that the boron complex can aggregate GlcN. Additionally, increased hydrogen-bonding interactions between the NH of GlcN and both the diol unit and B–OH of the Ery-BA complexes were also observed. As we have previously observed for other chitosan-based bioplastics, both aggregation and hydrogen-bonding with the NH functionality are keys to improving material properties. While previous studies have utilized boron-based species to manipulate a material’s properties by covalently cross-linking the polymer chains, the work described here focuses on the use of boric acid to alter the binding face of small molecule additives and thus modify its molecular-level interactions with the polymer. These studies continue to support the importance of developing a molecular-level understanding when designing additives capable of tuning the properties of chitosan materials.

**Figure 1:**
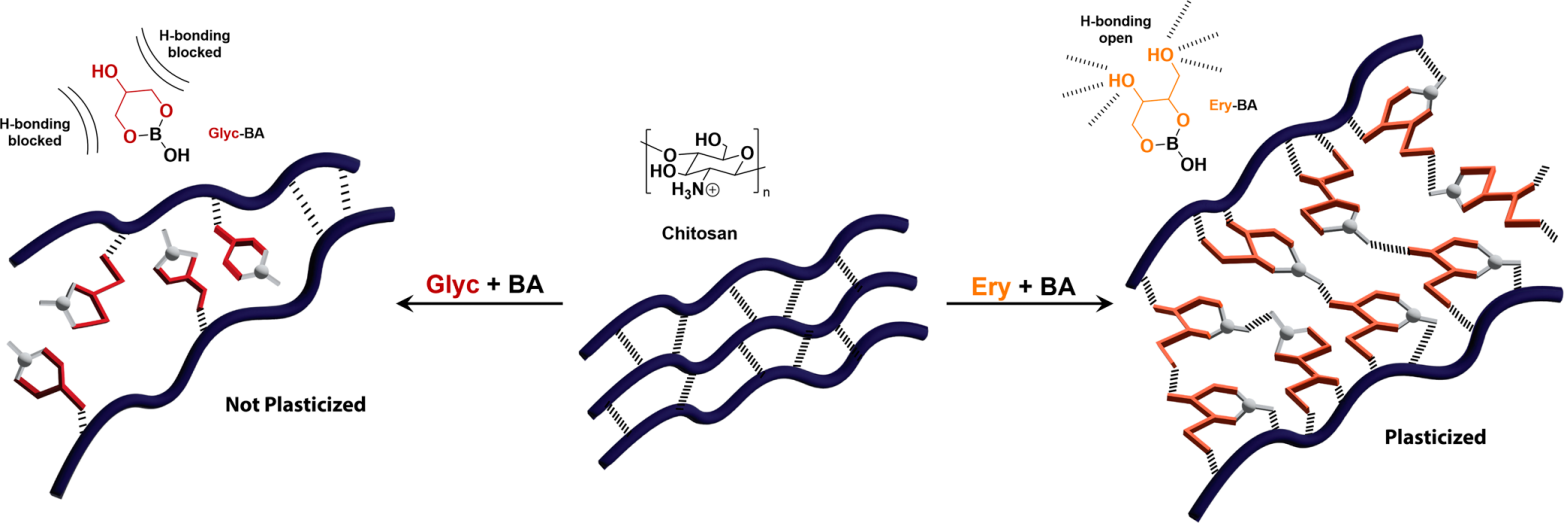
Effective plasticization of chitosan requires diol units capable of forming hydrogen bonds with the polymer chains. The formation of neutral boron complexes with glycerol (Glyc-BA) alters the binding face of Glyc, blocking hydrogen-bonding interactions and turning plasticization “off”. When neutral boron complexes of erythritol (Ery-BA) are formed, free diol units remain available for hydrogen-bonding leaving plasticization “on”.

## Materials and Methods

### Materials

Chitosan with a degree of deacetylation DD = 75–85% and a molecular weight range of 50,000–190,000 (low molecular weight), d-glucosamine hydrochloride (≥99%), *meso*-erythritol (≥99%) and DMSO-*d*_6_ (d,99.9%) were purchased from Sigma-Aldrich and used as received. Boric acid (ACS Certified), glycerol (ACS Certified), and glacial acetic acid (ACS Certified) were used as received from Fisher Scientific. Acetic acid solutions (1%) were obtained by diluting 1.0 g of glacial acetic acid with 99.0 g of deionized water. Chitosan stock solution (2 wt %) was prepared by rapid addition of chitosan (20.0 g) to stirring 1% acetic acid (980 mL) in an Erlenmeyer flask. The solution was left to stir for 2 days at room temperature until completely homogeneous. Stock solutions of *meso*-erythritol and boric acid (200 mM) were also prepared in 1% acetic acid for film preparation or in DMSO-*d*_6_ for NMR studies.

### Preparation of CER Films

Precast solutions were prepared by mixing the chitosan stock solution (10 mL) with increasing volumes of the erythritol stock solution (2.5 mL, 5 mL, 10 mL) and 1% aqueous acetic acid to reach a total volume of 20 mL. The resulting solutions contained 1 wt % chitosan with increasing concentrations of erythritol (25 mM, 50 mM, and 100 mM respectively). The precast solutions were allowed to stir at room temperature for 1 h. Precast solutions (5 mL) were dropcast into polystyrene dishes and allowed to dry in a 60 °C oven overnight.

### Preparation of CER-BA Films

Erythritol-boric acid containing chitosan films were prepared by first generating erythritol-boric acid stock solutions in 1% acetic acid. The erythritol concentration in each stock solution was held constant (50 mM) while the boric acid concentration was varied (25 mM, 50 mM, and 100 mM). Film solutions were then prepared by mixing the appropriate erythritol-boric acid stock solution (10 mL) with equal volumes of the 2 wt % chitosan stock solution (10 mL) to yield final concentrations of 1% chitosan, 25 mM erythritol and 12.5 mM, 25 mM or 50 mM boric acid, respectively. All film solutions were stirred at room temperature for 10 min to preserve the neutral boron complex formation in the precast solution. All films were dropcast into polystyrene dishes using 5 mL of the prepared solutions. The films were dried at 60 °C overnight. The same procedure was used to prepare films CER50-BA25-50. CER50-BA100 and CER100-BA25-200 films were prepared in a similar fashion, erythritol-boric acid stocks were prepared using the appropriate amount of solid boric acid due to the increased concentration required in these solutions.

### Characterization Methods

Attenuated total-reflection Fourier transform infrared (ATR-FTIR) spectroscopy of all films were performed on a Thermo Scientific Nicolet iS10 spectrometer, equipped with a SMART iTX attenuated total-reflection (ATR) accessory. The FTIR spectra were recorded from 400 to 4000 cm^–1^ with a resolution of 4 cm^–1^. Thermal gravimetric analysis (TGA) was performed using a TA Instruments Discovery TGA-550. Samples were heated from 25 to 500 °C with a heating rate of 10 °C/min. All TGA were collected under N_2_ gas with a flow rate of 40 mL/min. Kinetic analysis was performed by running the TGA at four ramp rates (2.5 °C/min, 5 °C/min, 10 °C/min, and 15 °C/min) up to 600 °C under N_2_ atmosphere. Samples were kept at similar mass ranges (∼2 mg) in order to limit the variability in the measurements. Differential scanning calorimetry (DSC) measurements were performed using a TA Instruments Discovery DSC-250. Films were crushed prior to transfer into the sample pans. Samples were run in aluminum pans and measurements were carried out under nitrogen gas flow. The samples were first equilibrated to −60 °C, then heated to 160 °C at a rate of 10 °C/min. After heating, the sample was cooled to −60 °C at the same rate. The mechanical properties were measured using a TA Instruments DHR-20 rheometer equipped with an ETC and film tension geometry. Axial strain sweeps were performed at 1 Hz and varying strain from 0.01 to 0.1%. Frequency sweeps were performed at 0.05% strain from 0.1 to 10 rad/s with 10 points per decade. The temperature was maintained at 25 °C with a gap width of 2960 nm for all measurements. Scanning electron microscopy (SEM) images were obtained using a Zeiss Sigma 300 VP field emission scanning electron microscope.

Nuclear magnetic resonance (^1^H, ^11^B, and ^13^C NMR; COSY, HSQC, HSQC-TOCSY, and NOESY; see Figures S1–S13, S24 and S25 for full spectra) spectra were obtained using a Bruker Avance DPX-400 NMR or a Bruker AVANCE II Ultrashield 600 MHz spectrometer in DMSO-*d*_6_. Chemical shifts are reported in ppm downfield from tetramethylsilane (δ scale) for ^1^H and ^13^C NMR and relative to BF_3_–OEt_2_ for ^11^B NMR spectra. NOESY measurements were recorded using the NOESYGPPHPP pulse protocol with a mixing time of 300 ms. Data were obtained with a 90° pulse of 10 μs and a relaxation delay of 2 s. A total of 24 scans with a spectral width of 3998 Hz in each dimension. Crosspeaks for NOE interactions and chemical exchange (EXSY) were distinguished by phase (i.e., NOE = negative, EXSY = positive).

## Results and Discussion

### Characterization of Erythritol-Boric Acid Complexes

To start, we evaluated the complexation of Ery with BA using 1D and 2D NMR techniques. ^1^H and ^11^B NMR titrations were performed with 50 mM Ery in DMSO-*d*_6_ and increasing amounts of BA (0–2.0 equiv) (Figures S1–S4). As anticipated, we observed a decrease in intensity for signals affiliated with free Ery with increasing amounts of BA and the emergence of several new sets of signals, which we assigned as Ery-BA complexes. Notably, the conversion of Ery to the Ery-BA complexes was found to be dependent on the concentration of water in the sample. This led to slight variations in the overall amount of Ery-BA complex formed, but did not impact the relative ratio of each isomer. From these spectra, it is obvious that Ery forms a number of unique neutral boron complexes composed of both monomeric (one polyol to one BA) and dimeric (one polyol to 2 BA) isomers. In an effort to best interpret these data and determine the major isomer, we turned our attention back to Glyc, a simpler triol which readily forms a mixture of two distinct neutral boron complexes. In our previous work, we reported the formation of a ∼50:50 mixture of the 1,2-Glyc-BA and 1,3-Glyc-BA complexes in DMSO-*d*_6_.^39^ Further analysis indicates the presence of several useful diagnostic signals for these isomers (Figures S5–S8, 2A). Specifically, the internal CH proton of the 1,2-Glyc-BA complex (H_c_) resolves as a multiplet at ∼4.26 ppm, while the 1,3-Glyc-BA isomer has a well-resolved multiplet at ∼3.78 ppm (H_d_) that is consistent with its internal CH proton. The diastereotopic protons on the adjacent CH_2_ (H_a_ and H_b_) in these ring systems are also distinct. In 1,2-Glyc-BA, these signals appear as doublet of doublets at ∼4.06 ppm and ∼3.79 ppm. In contrast, in the 1,3-Glyc-BA isomer, H_a_ and H_b_ are doublet of doublets at ∼3.92 ppm and ∼3.70 ppm. Using these diagnostic signals, we determined that two monomeric neutral boron complexes formed preferentially between Ery and BA (1,2-Ery-BA and 1,3-Ery-BA) with the 1,3-Ery-BA complex being the major isomer. Notably, little to no 2,3-Ery-BA isomer was observed (structure not shown). Shown in Figure 2B is a representative 1D NMR spectrum of 50.0 mM Ery with 1 equiv of BA in DMSO-*d*_6_. The major 1,3-Ery-BA isomer has the anticipated signals for the central CH (H_d_) at ∼3.68 ppm (m) and signals for the diastereotopic protons (H_a_ and H_b_) at ∼3.62 ppm (dd) and ∼3.91 ppm (dd). The 1,2-Ery-BA isomer has a diagnostic CH (H_c_) at ∼4.20 ppm, which resolves as a quartet. Though we would anticipate the protons on the CH_2_ of the ring (H_a_ and H_b_) to be diastereotopic and distinct, we observe only one signal for these protons that integrates to 2H at ∼3.99 ppm. The structures of these isomers were further confirmed using extensive 2D NMR experiments, and all ^1^H and ^13^C NMR peaks were assigned (Figures S9–S13). All additional signals in the ^1^H NMR spectrum were attributed to minor Ery-BA isomers.

**Figure 2:**
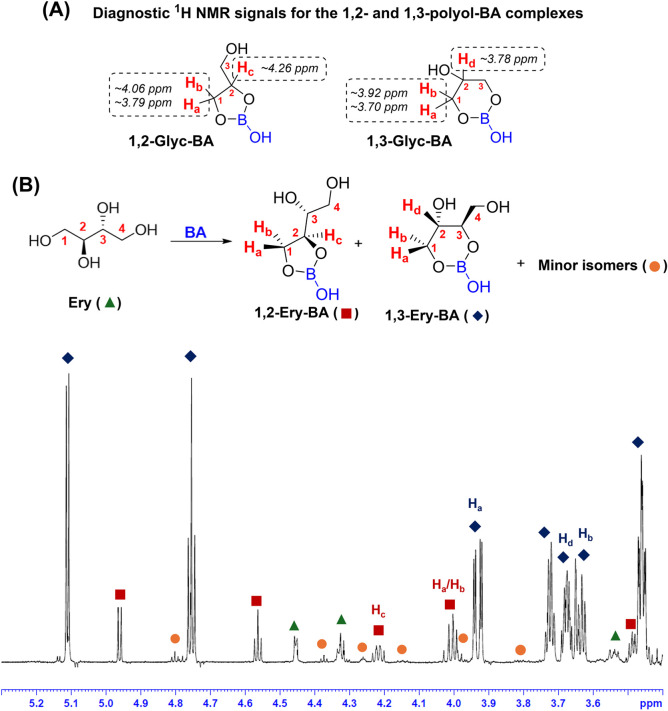
(A) Structures and diagnostic ^1^H NMR chemical shifts for the neutral boron complexes formed with Glyc (1,2-Glyc-BA and 1,3-Glyc-BA). (B) Proposed complexation reaction and labeled ^1^H NMR spectrum of 50.0 mM Ery with 1 equiv BA in DMSO-*d*_6_ recorded at 600 MHz (14.1 T).

### Film Preparation and Structural Characterization

After confirming and characterizing the Ery-BA complexes formed, we then incorporated these neutral boron complexes into chitosan films at various concentrations to understand the impact these structures have on the material properties. We were particularly interested in how the remaining free diol units interact with the chitosan chains and how those interactions impact thermal, mechanical and morphological properties.

To prepare the films, Ery-BA complexes were formed in 1% acetic acid using 50 mM erythritol and boric acid (25 mM, 50 mM, and 100 mM). The Ery-BA stock solutions were then combined in equal volumes with 2% chitosan in acetic acid before films were cast and dried in a 60 °C oven overnight. Isolated films CER25-BA12.5, CER25-BA25 and CER25-BA50 each contained 1% chitosan and 25 mM erythritol with 12.5 mM, 25 mM or 50 mM boric acid, respectively. Two additional series of CER-BA films (CER50-BA25-100 and CER100-BA50-200) were prepared with increasing concentration of erythritol (50 mM and 100 mM) while maintaining the relative equivalents of boric acid (0.5 equiv, 1.0 equiv, and 2.0 equiv). The equivalents of boric acid (0.5 equiv, 1.0 equiv, and 2.0 equiv) were chosen based on the molecular-level NMR studies to investigate the impact of the ratio of free Ery to Ery-BA complex on the material properties.

ATR-FTIR spectra were used to structurally characterize the interactions between chitosan and the neutral boron complexes. We have previously reported in-depth spectroscopic studies into the molecular-level interactions necessary to plasticize chitosan films.^39–41^ Control films of pure chitosan (PCF) and chitosan with boric acid (CBA) have been previously published and indicated that boric acid alone has minimal interactions with chitosan.^39^ All CER-BA films displayed evidence for free boric acid in the films at ∼1706 cm^–1^ (B–O–H) as well as the presence of the neutral boron complexes with a B–O stretch that grows in at ∼1485 cm^–1^ (Figures S14 and S15).^41–43^ Previously, we have reported on the importance of the amide II region of the IR spectra (∼1680 cm^–1^ to 1500 cm^–1^) as a tool for understanding the interactions between polyols and chitosan. Specifically, the interactions with the NH moiety which shifts to higher wavenumber in the presence of plasticizer.^40,41^ This shift is reduced in the presence of boric acid for Glyc-containing films by ∼17 wavenumbers. A similar shift is also observed for CER-BA films; however, the magnitude of this shift is significantly reduced to ∼5 wavenumbers. This shift remains nearly constant even with increasing concentration of boric acid (Figure S14). The reduced impact of the added boric acid with Ery can be rationalized by the presence of the free diol units in the Ery-BA complexes. Our previous studies have shown that diols readily interact with chitosan chains.^41^ In contrast, with Glyc-BA complexes, only an isolated OH unit remains available when BA is bound. The impacts of these binding face alterations will be expanded upon in the molecular-level NMR studies detailed at the end of this paper.

### Thermal and Mechanical Characterization

Chitosan is known to undergo three stages of decomposition in the presences of O_2_. These are loss of water at lower temperature, followed by the most significant mass loss (∼50%) which can be attributed to depolymerization of the polymer chains via cleavage of the glycosidic linkages and deacetylation, and last above 400 °C, the thermal destruction of the pyranose rings and any residual carbon.^44–49^ However, experiments performed under N_2_ do not show the final decomposition step, leaving some mass remaining even up to temperatures above 600 °C. All of the experiments detailed here have been performed under N_2_.^37,39,50^ Previous reports have indicated that the addition of polyols alone have an insignificant impact on the thermal stability of chitosan films.^37^ In CER25, CER50, and CER100 films three thermal transitions (*T*_1–3_) can be observed in the thermogravimetric analysis (TGA) and differential thermal analysis (DTG), which can be attributed to the loss of water (*T*_1_) and Ery (*T*_2_) from the films followed by chitosan decomposition (*T*_3_) (Figure 3, Table 1). Slight increases in *T*_2_ were observed with increasing Ery concentration. Compared to PCF, minimal changes to *T*_3_ were observed from 267 °C to ∼240 °C for all of the films with increasing Ery concentration confirming that the polyol alone has a minimal impact on the thermal stability of chitosan (Figures 3A–C, S16). However, our findings have indicated a notable improvement in thermal stability with increasing BA concentration.^39^ CER-BA systems also display three thermal transitions (*T*_1–3_) in the TGA and DTG curves (Figures 3A–C, S16).

**Figure 3:**
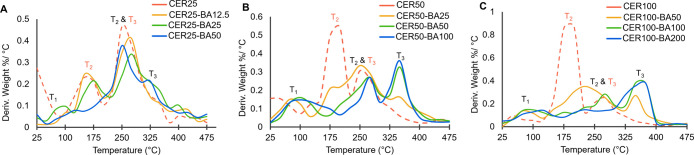
DTG curves of CER25 (A-red), CER50 (B-red) and CER100 (C-red) with increasing equivalents of BA (0.5 equiv-gold, 1.0 equiv-green, 2.0 equiv-blue). Dashed lines indicate materials with no BA. *T*_1–3_ notations highlight the loss of water (*T*_1_), Ery (*T*_2_), and decomposition of chitosan (*T*_3_). *T*_2_ and *T*_3_ transitions labeled in red indicate the transitions without the addition of BA.

Similarly to CER films, *T*_1_ can be attributed to the loss of water in the material; for all concentrations of Ery and BA, this transition occurs between 50 and 70 °C. Interestingly, the water content in the films remains reasonably constant between 6 and 10% for CER50-BA and CER100-BA films with increasing concentration of boric acid. This is in contrast to the Glyc-BA containing materials which displayed a decrease in water content with increasing boric acid concentration.^39^ This can again be rationalized because of the presence of free diol units on the Ery-BA complexes, which partially retain the hydrophilic nature of the polyol. In Glyc-containing systems this correlates with the ability of films to retain moisture.^37^

Following the loss of water, *T*_2_ is observed between 96 and 254 °C, which can be attributed to the loss of Ery from the films. At low concentrations of Ery in the films, *T*_2_ temperatures are lower than that of the decomposition temperature for pure Ery, which indicates that Ery–Ery interactions are stronger than the Ery-chitosan intermolecular interactions (Figure S16). The increased temperature of *T*_2_ at higher concentrations can be attributed to the increased presence of Ery–Ery interactions within the plasticized material. In CER-BA films with substoichiometric amounts of BA, two *T*_2_ transitions can be observed in the DTG curves (Figure 3). The *T*_2_ at lower temperature would indicate loss of uncoordinated Ery from the films while the *T*_2_ at higher temperature would indicate the loss of Ery from the Ery-BA complexes. The temperature of this transition increases with increasing boric acid concentration. The third thermal transition, *T*_3_, occurs between 240 and 356 °C and is indicative of the complex decomposition involving both the acetylated and deacetylated units of the polymer structure.^44,46,48,49^ For the films with lower concentrations of Ery (CER25-BA12.5–50) *T*_3_ increases only slightly as the BA concentration is increased (240.5 °C, 249 °C, and 242 °C, respectively). However, when the amount of Ery in the films is increased to 50 or 100 mM (CER50-BA25-100 and CER100-BA50-200), increasing the concentration of boric acid in the system increases *T*_3_ to 232–342 °C and 336–354 °C for CER50-BA25-100 and CER100-BA50-200 films, respectively. This would suggest that at higher concentrations, the Ery-BA complexes are capable of interacting with the polymer chains in a significant way.

To further investigate the interactions responsible for changes to both *T*_2_ and *T*_3_, kinetic analysis was performed. TGA of all samples were run at four different temperature ramp rates (2.5 °C/min, 5 °C/min, 10 °C/min, and 15 °C/min). Two isoconversional methods, Ozawa-Flynn-Wall (OFW) and Kissinger-Akahria-Sunose (KAS), were used to fit the data^51,52^ in order to obtain the activation energy (*E*_a_) (Figure S17A,B). These isoconversional models described by the ICTAC have been previously used for dynamics analysis of chitin and chitosan under N_2_ and ambient conditions.^44,50^ The increased temperature of *T*_2_ in the presence of boric acid indicates the ability of BA to stabilize the plasticizer–polymer interactions. This can be further confirmed with kinetic analysis of these materials. The *E*_a_ for the loss of Ery, at mass loss fraction alpha = 0.3–0.5, from the material remains constant among all samples at 144.5 ± 16 kJ/mol while *T*_2_ increases (Figure S17A,B). This suggests that the stability of the interaction increases, while the mechanism of decomposition remains the same. Using the KAS model, one can plot ln­(beta/*T*_m_^2^) versus 1/Temperature to obtain the *E*_a_ of the transition. Where *T*_m_ is the temperature at the maximum rate of decomposition, which we have assigned to *T*_3_ and beta is the heating rate (Figure S17B). As we previously observed in the DTG curves, the *T*_m_ (*T*_3_) increases with increasing BA concentration. The average *E*_a_ of this decomposition across all films is 142.17 ± 17 kJ/mol which is in good agreement with the previously reported *E*_a_ for pure chitosan (146.50 kJ/mol)^44^ and is within the accepted error noted by the ITCAC of 5–20% (Figure S17B).^53^ These results indicate that the Ery-BA complexes are capable of stabilizing the chitosan as evidenced by the increases in *T*_3_ with increasing Ery-BA concentration, however they do not have an impact on the mechanism of decomposition as evidenced by lack of change in *E*_a_ of the various films.

Differential scanning calorimetry (DSC) was then used to obtain the glass transition temperature (*T*_g_) of the materials (Figure 4, Table 1). This is an important physical property of polymers because *T*_g_ can describe the extent of plasticization of the material, where a reduction in the *T*_g_ relates to an increase in plasticization and an increase in the flexibility of the material. As anticipated, the addition of Ery significantly lowers the *T*_g_ from 49.9 °C in PCF^39^ to 3.8 °C for CER25 (Figure S18).

**Figure 4:**
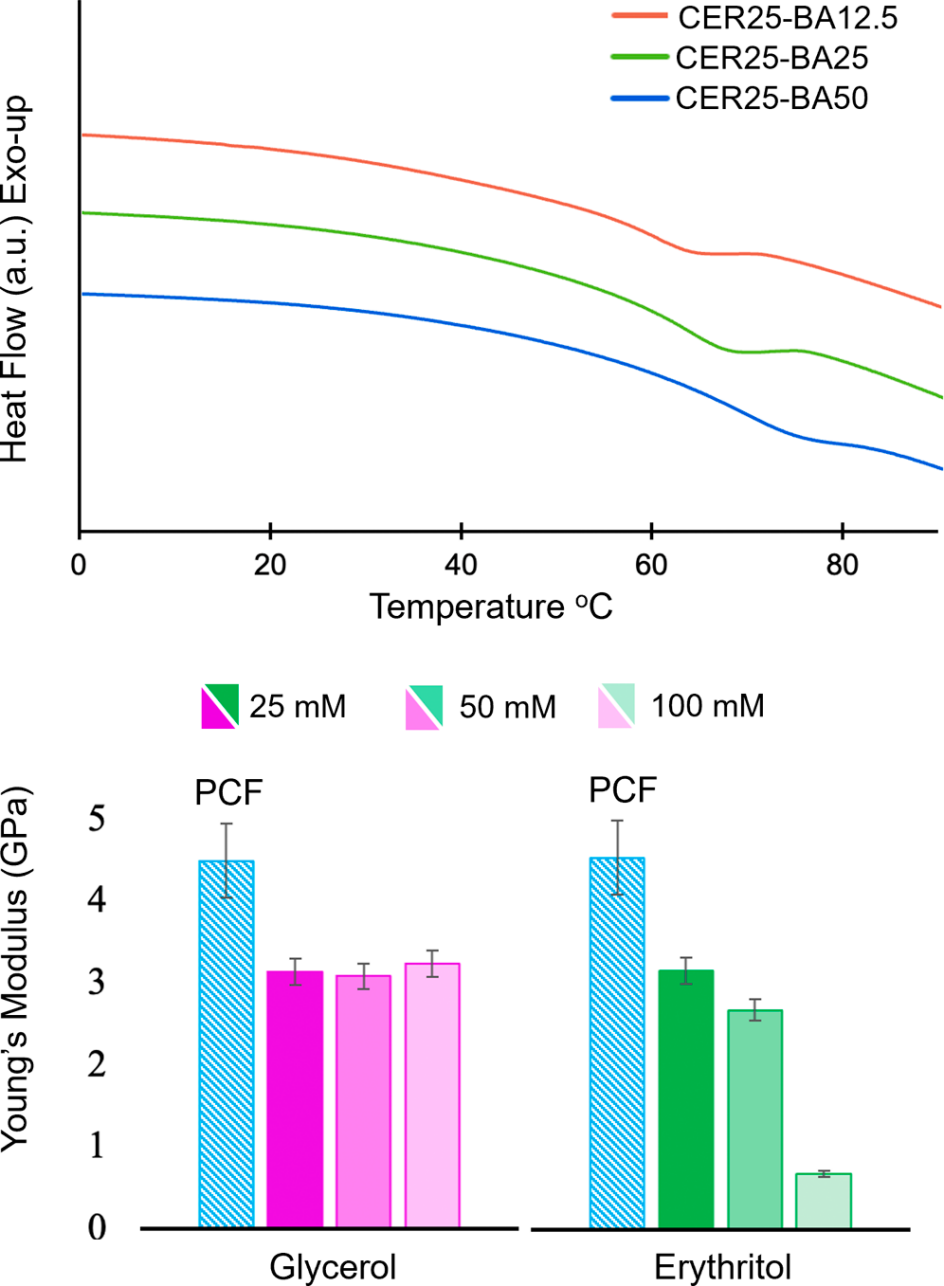
(Top) Section of the DSC thermograms of CER25-BA12.5 (red), CER25-BA25 (green), and CER25-BA50 (blue) highlighting the increase in *T*_g_ with increasing BA concentration. (Bottom) Plot of Young’s modulus of 25 mM, 50 mM, and 100 mM Glyc- and Ery-containing chitosan films with 1 equiv of BA (25 mM, 50 mM, 100 mM, respectively). Additional DSC and DMA data of all films can be found in Figures S18–S20.

**Table 1:** Combined Thermal and Mechanical Data for CER and CER-BA Films^a^

film	*T*_1_ (°C)	WL_1_ (%)	*T*_2_ (°C)	WL_2_ (%)	*T*_3_ (°C)	WL_3_ (%)	*T*_g_ (°C)	*E* (GPa)
CER25	13.5	10.9	134.1	17.8	240.1	37.5	3.8	1.65
CER25-BA12.5	16.4	1.1	137.0	14.2	240.5	33.1	55.7	1.50
CER25-BA25	56.9	4.2	147.3	12.3	249.2	29.3	58.7	3.13
CER25-BA50			95.6	4.8	242.4	36.4	64.1	5.44
CER50	19.0	10.8	172.7	33.3	240.3	21.6	–20.7	1.13
CER50-BA25	49.5	9.1	175.0	14.9	232.7	33.3	50.6	2.17
CER50-BA50	61.2	8.1	238.2	25.8	342.7	20.6	52.9	2.66
CER50-BA100	58.2	13.3	254.0	16.2	329.9	20.8	52.0	3.23
CER-100	15.9	6.1	167.6	49.6	246.3	18.5	–32.3	0.43
CER100-BA50	52.0	8.7	173.8	41.5	348.6	17.8	37.6	0.48
CER100-BA100	58.5	10.1	229.4	28.2	354.5	29.6	42.5	0.68
CER100-BA200	67.1	11.8	213.0	14.8	336.0	32.0	59.8	0.91

a Temperatures *T*_1_, *T*_2_, and *T*_3_ are the onset temperatures for the loss of water (*T*_1_) and erythritol (*T*_2_) from the films and the decomposition of chitosan (*T*_3_). WL_1‑3_ corresponds to the percent mass loss during each of these transitions. All values have an error of less than 5%.

Increasing the concentration of Ery continues to lower the *T*_g_ to −20.7 °C and −32.3 °C for CER50 and CER100 respectively (Figure S16). CER100 does display a *T*_m_ at ∼120 °C, which can be attributed to excess Ery in the system as was observed in the TGA (Figure S18). The addition of BA increases the *T*_g_ for all concentrations of Ery. CER25-BA12.5-50, CER50-BA25-100, and CER100-BA50-200 show ranges of *T*_g_ of 56–64 °C, 51–52 °C, and 38–60 °C, respectively (Figures 4 and S19). When compared to Glyc-BA containing films, which have *T*_g_ values ranging from 70 to 77 °C (50 mM Glyc, 25–100 mM BA) all of the CER-BA systems show lower *T*_g_ values.^39^ This indicates that although increasing the concentration of BA indeed reduces the plasticizing capability of Ery, the free diols remaining in the Ery-BA complexes can maintain interactions with the chitosan chains. For example, CER100-BA25 and CER100-BA50 exhibit glass transition temperatures below that for PCF while showing a significant increase in decomposition temperature of the material thereby maintaining flexibility and increasing thermal stability (Table 1, Figure S19).

The viscoelastic behavior and stiffness of these films were investigated with dynamic mechanical analysis (DMA) (Figures 4, S20). A decrease in the Young’s modulus for CER25-100 (1.65, 1.13, and 0.43 GPa, respectively) was observed when compared to PCF (4.50 GPa).^41^ This is consistent with Ery acting as a plasticizer resulting in an increase in the flexibility of the films. As was previously observed for Glyc-BA containing systems, as the concentration of BA was increased and more neutral boron complexes were formed, the Young’s moduli of the materials increased (Table 1). In films containing 50 mM Glyc with increasing BA (CG50-BA25, CG50-BA50, and CG50-BA100), the range of Young’s modulus values reported were 2.25–8.00 GPa.^39^ At the same concentrations (CER50-BA25-100) the materials containing Ery-BA show a decrease in Young’s modulus with values ranging from 2.17 to 3.23 GPa (Table 1). In comparing Glyc-BA and Ery-BA containing films with 1 equiv of BA and increasing polyol concentration, an interesting trend emerges (Figure 4). In the Glyc-BA systems (CG25-BA25, CG50-BA50, and CG100-BA100) the Young’s modulus remains constant at ∼3 GPa even at high concentrations of Glyc (CG100-BA100). In contrast a significant decrease in the Young’s modulus is observed for Ery-BA containing film when the concentration of polyol is increased from 3.13 GPa in CER25-BA25 to 0.68 GPa in CER100-BA100. This result highlights the impact of the remaining free OH units in the two different neutral boron complexes. At this 1:1 ratio of polyol/BA, increasing concentration of the Glyc-BA complex only increases the concentration of the singular free OH unit of the complex, while increasing the concentration of the Ery-BA complex results in an increase in the number of diol units, which are capable of plasticizing interactions.

### Morphological Characterization

Cross-sectional morphologies were obtained using scanning electron microscopy (SEM). Surface images are less informative in these systems when drawing connections between morphology and physical properties. We have previously observed that the cross-section of PCF exhibits a very heterogeneous internal structure with various size pores. This internal structure changes drastically to a more uniform morphology in the presence of Glyc (CG), while a reduction in pore-like structure and a more ordered morphology was observed for CG-BA films (Figure S21).^39^ For CER25-100, we can again observe a reduction in the pore-like structures present in PCF (Figure 5A–C). Additionally, the cross-section appears to become more uniform in nature as the concentration of Ery increases. In contrast to CG-BA films, CER-BA films do not show an increase in order, but rather appear to have an increase in smoothness even at high concentrations of boric acid (Figure 5D–F, S22 and S23). The drastic difference between the ordered nature of the CG-BA films and the smoothness of the CER-BA films correlates well with the physical and mechanical properties previously described, supporting the impact of the free diol of the neutral Ery-BA complexes.

**Figure 5:**
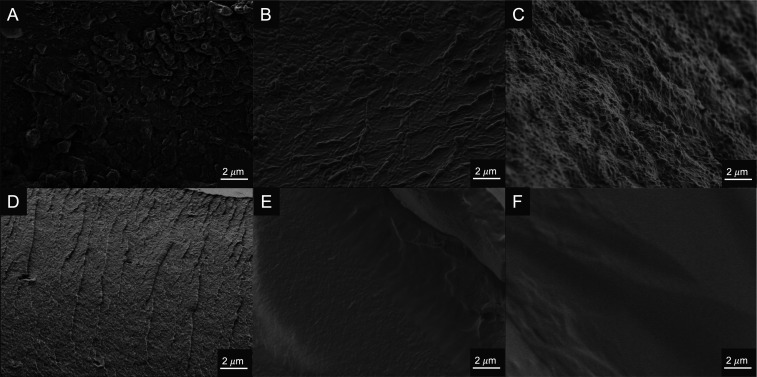
Cross-sectional SEM images of (A) CER25, (B) CER50, (C) CER100, (D) CER25-BA100, (E) CER50-BA100, and (F) CER100-BA100. All images are shown at 10k× magnification. Images A–C show an increase in the uniformity and smoothness of CER films with increasing Ery concentration. Images D–F display an increase in smoothness of CER-BA films, all films contain 100 mM BA with increasing concentration of Ery.

### Characterization of Molecular-Level Interactions between GlcN and Erythritol-Boric Acid Complexes

Lastly, in an effort to rationalize the observed material and morphological properties of CER-BA films, we turned our attention back to molecular-level NMR studies. Our previous work has demonstrated that GlcN, the monomeric repeat unit of chitosan, is an excellent model for evaluating the intra- and intermolecular interactions within chitosan films.^39–41^ In particular, nuclear Overhauser effect spectroscopy (NOESY) has been a helpful tool for probing through space and chemical exchange (EXSY) interactions between protons that are in close proximity. Our previous spectroscopic studies with Glyc and other polyols have indicated that effective plasticization of chitosan is the result of direct and specific hydrogen-bonding interactions between the plasticizer and the polymer. We found that Glyc has a notable preference for the NH functionality of GlcN, an interaction also observed in the chitosan films. Additionally, we found that Glyc was capable of disrupting intramolecular interactions, while promoting GlcN–GlcN aggregation. Based on these findings, we concluded that interactions between the NH of GlcN and the polyol and increased GlcN–GlcN aggregation correlates with better plasticization in chitosan. With this in mind, we sought to evaluate the differences in the molecular-level interactions between GlcN and either Glyc or Ery when BA is introduced. Shown in Figure 6 are selected cross sections of the 2D NOESY spectra at 3.0 mM GlcN in DMSO-*d*_6_ with 1.0 equiv of polyol and 1.0 equiv of BA (for full spectra, see Figures S24 and S25). A concentration of 3.0 mM GlcN was chosen as it corresponds with GlcN in an “isolated” state and therefore allows us to evaluate GlcN-diol interaction and polyol-promoted GlcN–GlcN association. Notably, in the presence of Glyc-BA complexes, we observe a negative NH–CH1 crosspeak for GlcN, consistent with an NOE interaction and the protons being in close proximity. Additionally, two small positive crosspeaks are observed for NH-BA and NH-Glyc, indicating there are weak hydrogen-bonding interactions between the NH of GlcN and both BA and free Glyc. Importantly, we observe no evidence of intermolecular interactions between the neutral boron complexes of Glyc and GlcN. In contrast, with Ery-BA, we observe distinct interactions between GlcN and the Ery-BA complex, as well as a number of GlcN–GlcN interactions. In this case, the NH of GlcN has strong positive crosspeaks with OH1 (NH–OH1), OH_4_, (NH–OH_4_) and OH_6_ (NH–OH_6_), consistent with intermolecular GlcN–GlcN interactions and aggregation. Furthermore, though we still observe GlcN interactions with free BA and free polyol, there are additional positive crosspeaks between the NH of GlcN and all signals of the 1,3-Ery-BA complex. This indicates that complexation of Ery with BA does not diminish its capacity to form hydrogen bonds with GlcN and is consistent with the diol unit of Ery-BA complex plasticizing chitosan.

**Figure 6:**
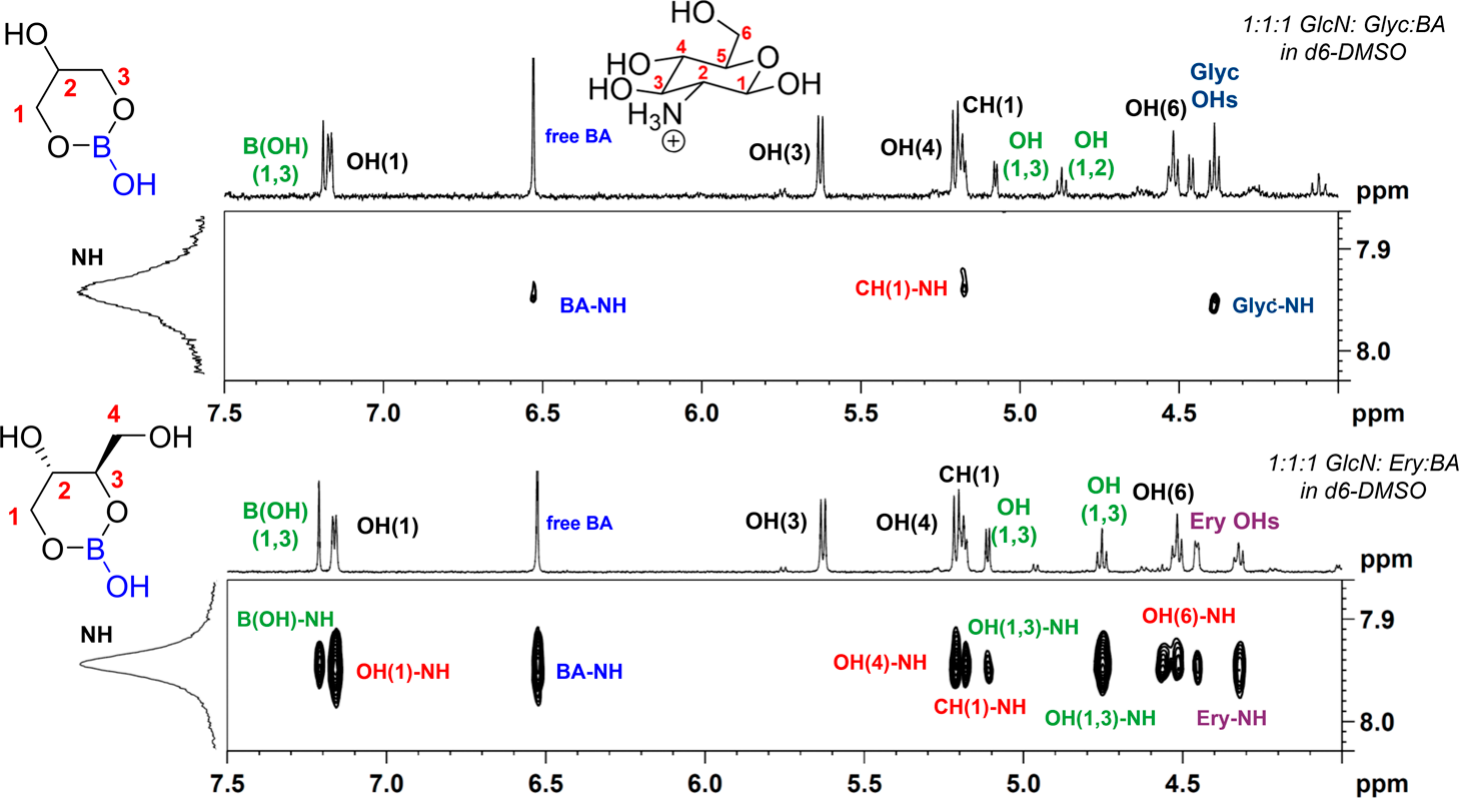
Representative cross sections of the 2D NOESY spectra of the NH of GlcN at 3.0 mM GlcN, 3.0 mM BA and 3.0 mM Glyc (top) or 3.0 mM Ery (bottom) in DMSO-*d*_6_ recorded at 400 MHz (9.4 T).

## Conclusions

In conclusion, we have investigated the ability of neutral Ery-BA complexes to plasticize chitosan and yield materials with flexibility and increased thermal stability. Through extensive 1D and 2D NMR experiments we have been able to identify the preference for the formation of 1,3-Ery-BA complexes, which provides free 1,3-diol binding units that can form hydrogen bonds within chitosan. We characterized the films structurally, thermally, mechanically, and morphologically to get a full picture of how the material properties were impacted by the presence of these residual diol units. The presence of the neutral boron complexes within the polymer films was confirmed using IR spectroscopy. As the concentration of BA in the materials is increased, a decrease in the shift of the NH functionality is observed indicating a reduction in the hydrogen-bonding interactions between Ery and chitosan. However, because of the remaining free diol units in the Ery-BA complexes, the magnitude of this shift is decreased when compared to Glyc-BA containing materials. An increase in the thermal decomposition temperature of the CER-BA materials with increasing BA concentration was observed, with the highest decomposition temperatures being obtained in films with the highest concentration of the Ery-BA complexes. Kinetic analysis revealed that Ery-BA complexes increase the thermal stability of chitosan, while the mechanism of decomposition remains unchanged. This increase in thermal stability was also observed while maintaining reasonably low *T*_g_ and Young’s moduli when compared to PCF. Morphologically, materials containing the Ery-BA complexes exhibited smooth internal cross sections and a reduction in the heterogeneity that was observed in PCF. These observed morphologies were in agreement with both the thermal and mechanical data described. These combined data are beneficial in the future design of chitosan-based materials in which both increased thermal stability and flexibility are required, such as for food packaging or biomedical applications.

Further studies into the molecular-level interactions between GlcN and these neutral boron complexes were also conducted. These data highlight the difference between Glyc-BA and Ery-BA in their ability to aggregate GlcN. Our previous work has shown that increased aggregation translates to improved material properties in the polymeric systems. Our findings reported herein have utilized our previous reports, which highlight the necessity of specific molecular-level interactions between the plasticizer and the polymer chains, to achieve effective plasticization. We have shown that plasticization can be maintained with neutral boron complexes if diol binding units remain accessible. Currently, we are continuing to explore the role of plasticizer structure and the impacts of boric acid in chitosan films. Specifically, we are investigating BA complexation with longer and more functionalized polyols and are evaluating the stereochemical variations of the diol or triol units in polyol-BA complexes. Additionally, we are expanding on our foundational studies to investigate the performance of these modified chitosan materials in environments relevant to food packaging and biomedical applications. The insights gained from these studies will allow for the development of customized plasticizers yielding the diverse material properties needed in bioplastics.

## Supplementary Material



## Notes

1D NMR spectra including ^11^B, ^1^H, ^13^C NMR of Ery-BA and Glyc-BA complexes. 2D NMR spectra including COSY, HSQC, and HSQC-TOCSY of Ery-BA and Glyc-BA complexes. ATR-FTIR spectra, TGA and DSC thermograms, DMA stress v strain plots, and SEM images of all CER and CER-BA films (PDF)

## Abbreviations

Glyc: glycerol
Ery: erythritol
BA: boric acid
ATR-FTIR: attenuated total reflectance Fourier transform infrared spectroscopy
TGA: thermogravimetric analysis
DTG: differential thermal analysis
OFW: Ozawa-Flynn-Wall
KAS: Kissinger-Akahria-Sunose
DSC: differential scanning calorimetry
NMR: nuclear magnetic resonance
DMA: dynamic mechanical analysis
SEM: scanning electron microscopy
NOSY: nuclear Overhauser effect spectroscopy
GlcN: d-glucosamine hydrochloride
CER: chitosan polymers containing erythritol, number following indicates erythritol concentration
CG: chitosan polymers containing glycerol, number following indicates glycerol concentration
CER-BA: chitosan polymers containing erythritol and boric acid, numbers following indicates erythritol and boric acid concentrations
CG-BA: chitosan polymers containing glycerol and boric acid, numbers following indicates glycerol and boric acid concentrations.
