# Supplementary material for: Modulating Thermal Stability and Flexibility in Chitosan Films with Neutral Polyol-Boric Acid Complexes
Source: Biomacromolecules. 2025 Jun 26;26(7):4174–83. doi: 10.1021/acs.biomac.5c00177 (PMC12264955; doi:10.1021/acs.biomac.5c00177)
Supplement: Supplementary file 1 [file bm5c00177_si_001.pdf]

Supporting Information

Modulating Thermal Stability and Flexibility in Chitosan Films with Neutral Polyol-Boric Acid Complexes

Olivia E. Coer, Brandy L. Davidson, Brycelyn M. Boardman, and Gretchen M. Peters**

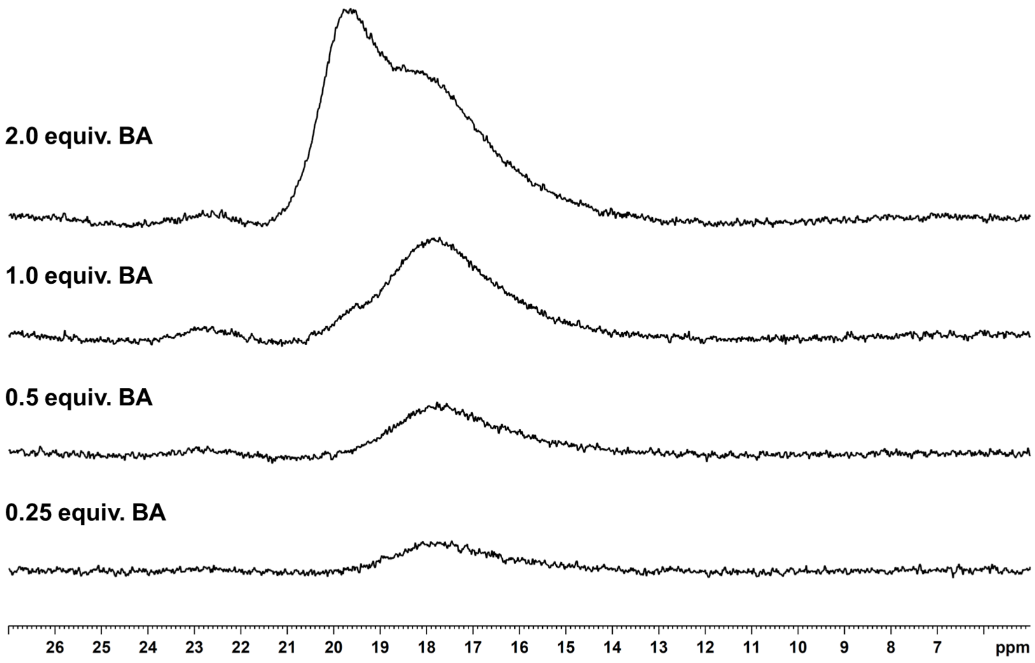

SI-Figure 1. ^{11}B NMR titration of 50.0 mM Ery with 0.25 - 2.0 equivalents BA in d6-DMSO recorded at 400 MHz (9.4 T).

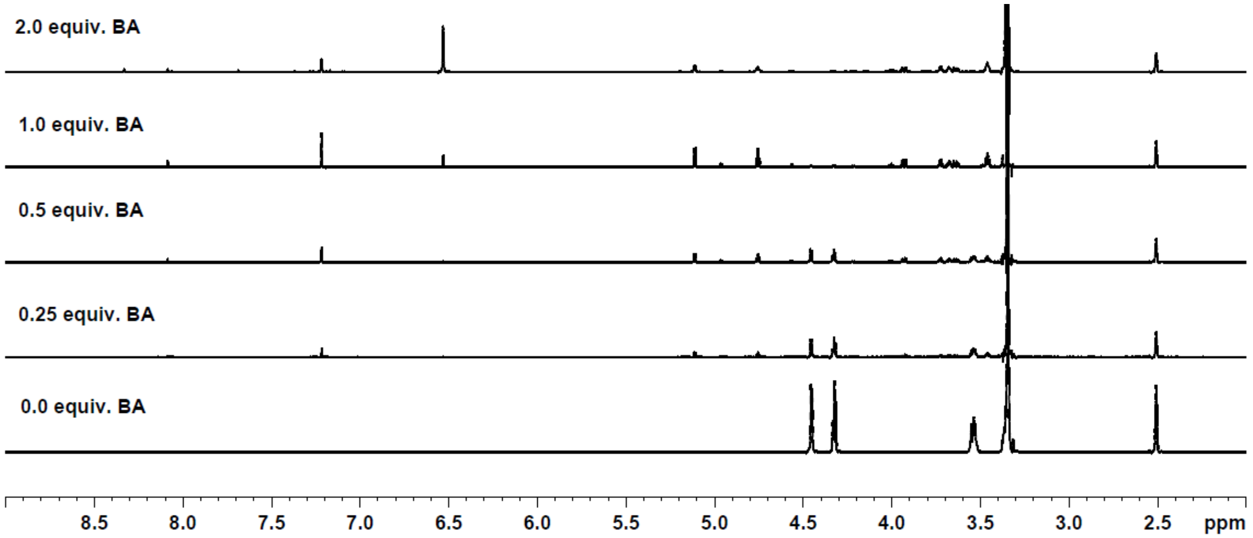

SI-Figure 2. Full ^{1}H NMR spectra of 50.0 mM Ery and 0 - 2.0 equivalents BA in d6-DMSO recorded at 400 MHz (9.4 T).

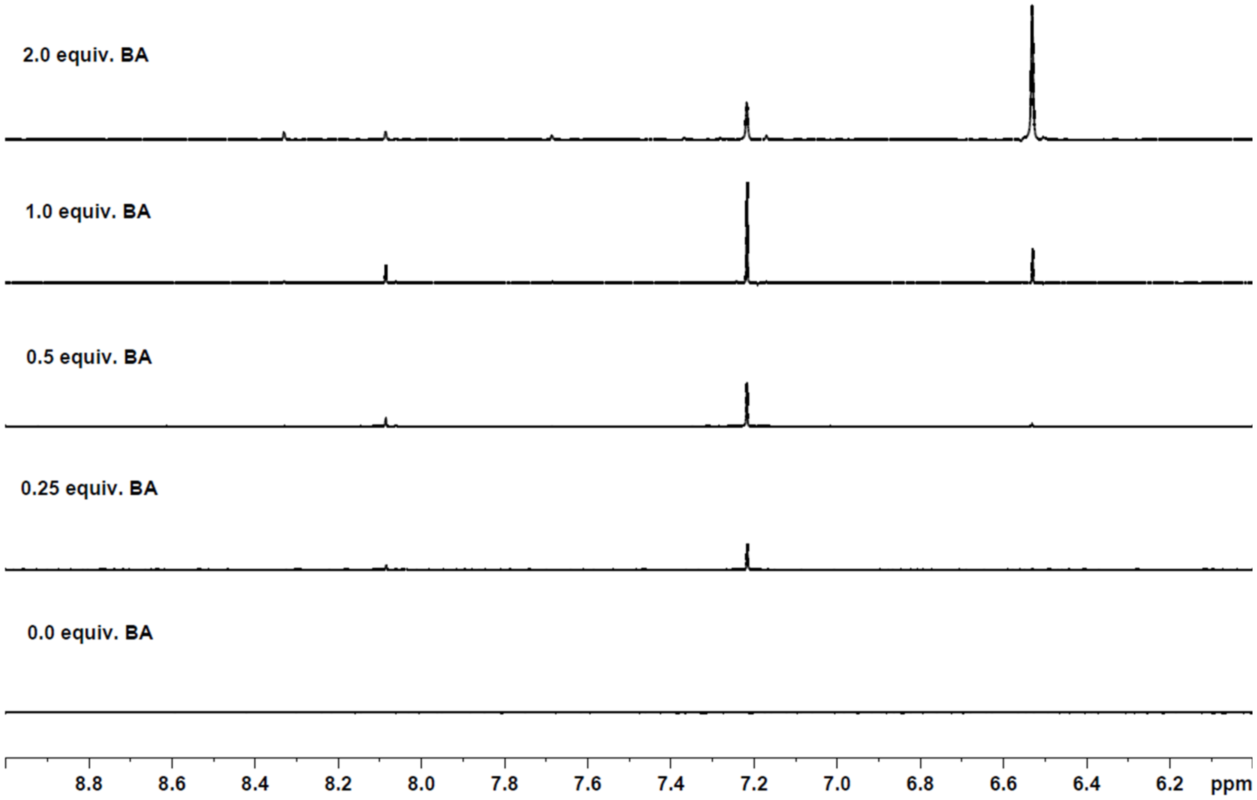

SI-Figure 3. Zoomed portion of the ^{1}H NMR spectra (9 - 6 ppm) of 50.0 mM Ery and 0 - 2.0 equivalents BA in d6-DMSO recorded at 400 MHz (9.4 T).

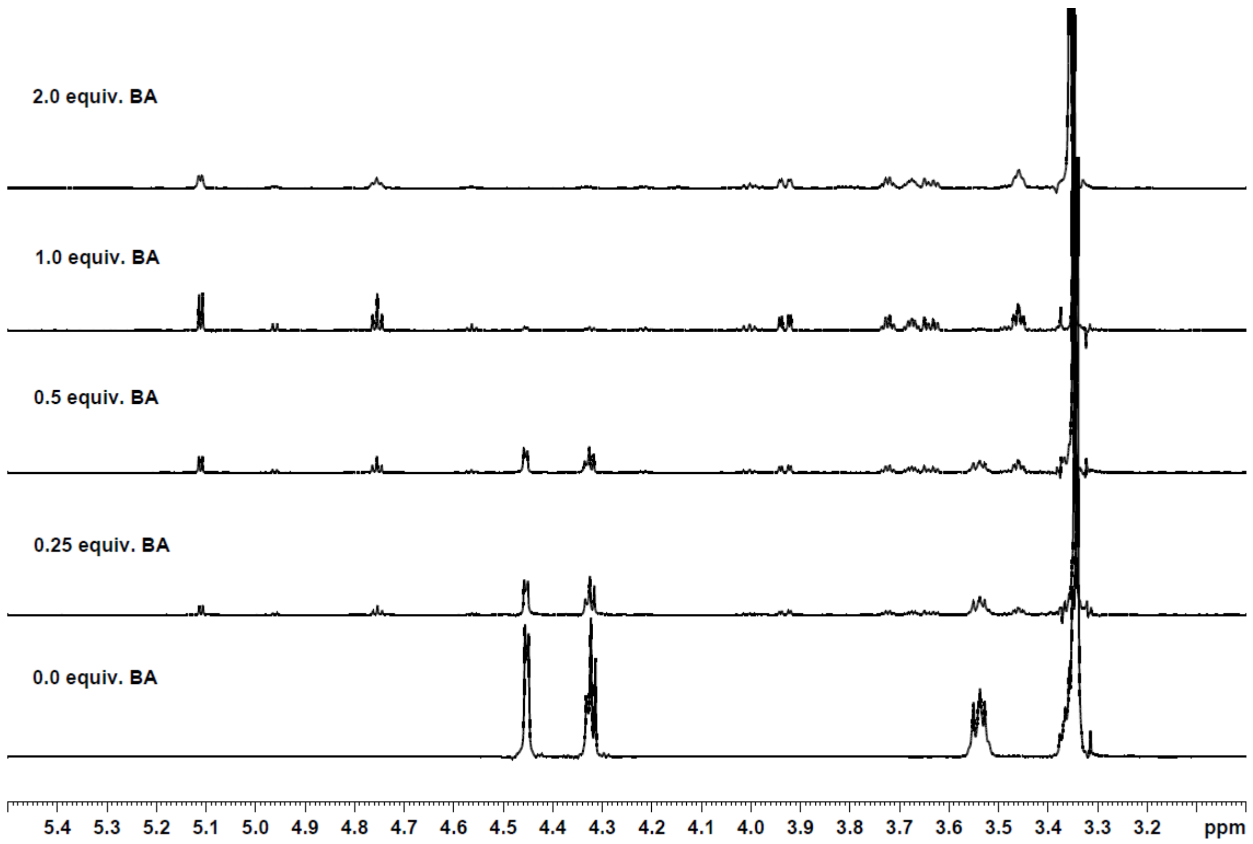

SI-Figure 4. Zoomed portion of the ^{1}H NMR spectra (5.5 - 3 ppm) of 50.0 mM Ery and 0 - 2.0 equivalents BA in d6-DMSO recorded at 400 MHz (9.4 T).

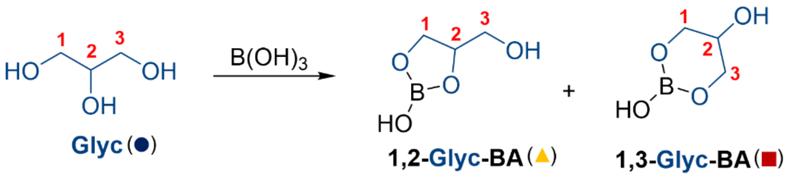

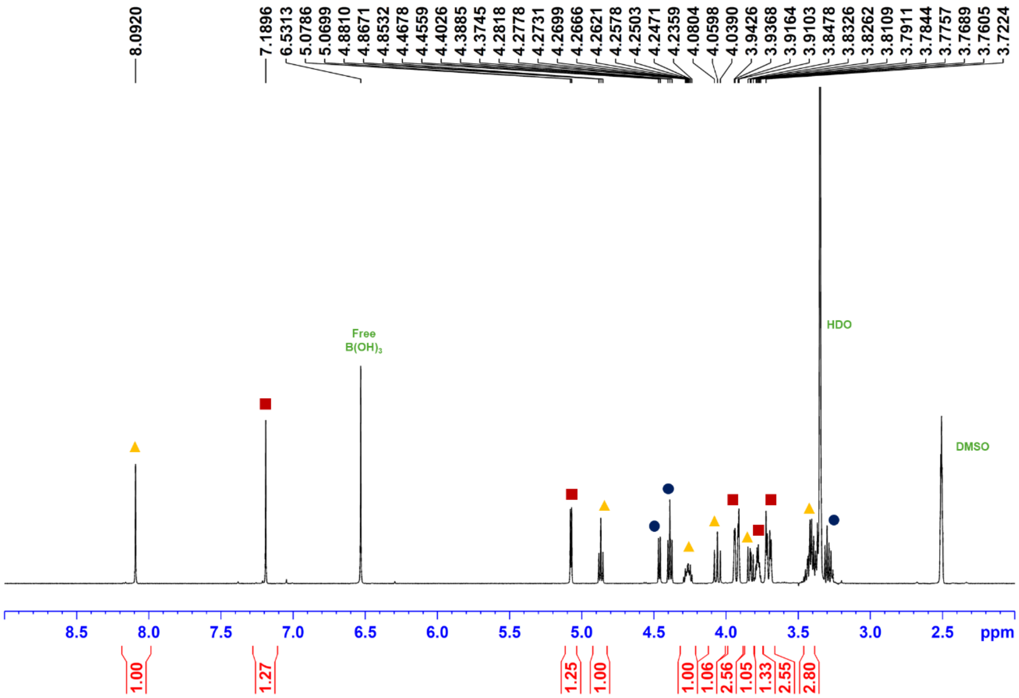

Isomer	Proton	δ (ppm)	Integration	Multiplicity
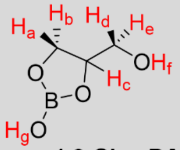 1,2-Glyc-BA (▲)	H_a	4.0598	1.0617	dd*
	H_b	3.7911	1.0457	dd
	H_c	4.2621	1.0000**	m
	H_d/H_e	3.4060	2.8016	m
	H_f	4.8671	0.9959	t
	H_g	8.0920	1.0009	s
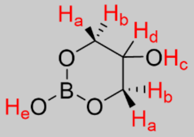 1,3-Glyc-BA (■)	H_a	3.9164	2.5556	dd
	H_b	3.6962	2.5537	dd
	H_c	5.0699	1.2482	d
	H_d	3.7757	1.3291	m
	H_e	7.1896	1.2653	s

*H_a appears as a triplet. **H_c of the 1,2-Glyc-BA isomer was set as 1.0000 for the integrations. H_d/H_e has significant overlap with the proton signals for free Glyc.

‡The stereochemistry of H_a/H_b and H_d/H_e has been arbitrarily assigned. Stereochemistry at C2 has been omitted for clarity.

SI-Figure 5. ^{1}H NMR spectrum and spectral details of 50.0 mM Glyc and 50.0 mM BA in d6-DMSO recorded at 400 MHz (9.4 T).

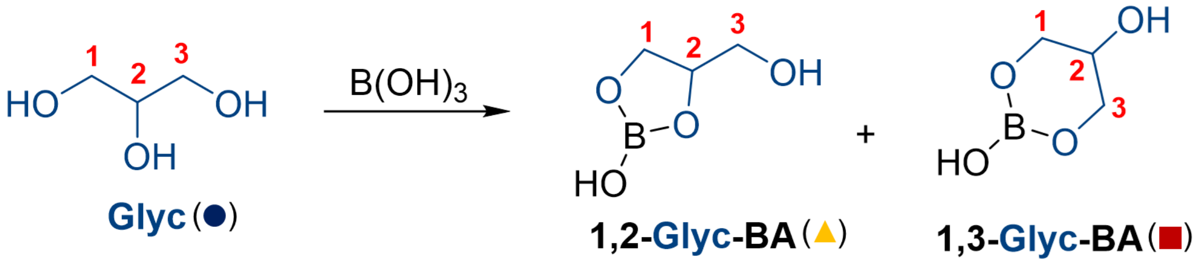

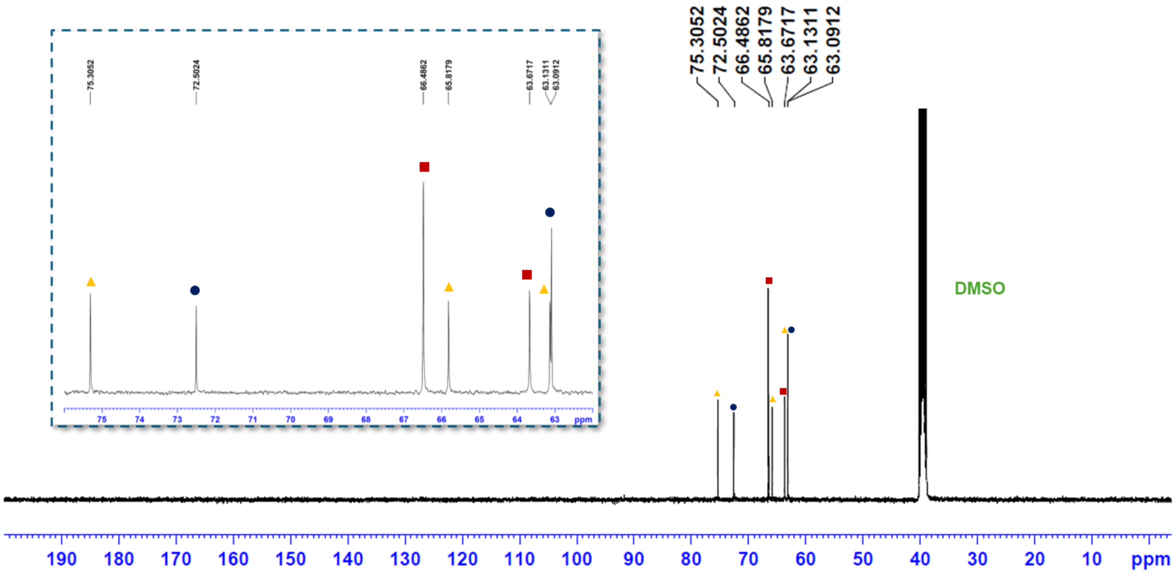

Isomer	Carbon	δ (ppm)
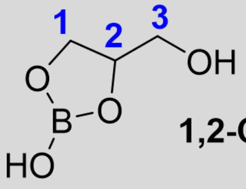 1,2-Glyc-BA (▲)	C1	65.8179
	C2	75.3502
	C3	63.1311
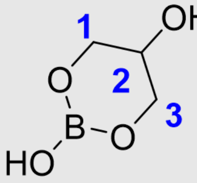 1,3-Glyc-BA (■)	C1/C3	66.4862
	C2	63.6717

Figure 6. ^{13}C NMR spectrum and spectral details of 50.0 mM Glyc and 50.0 mM BA in d6-DMSO recorded at 400 MHz (9.4 T).

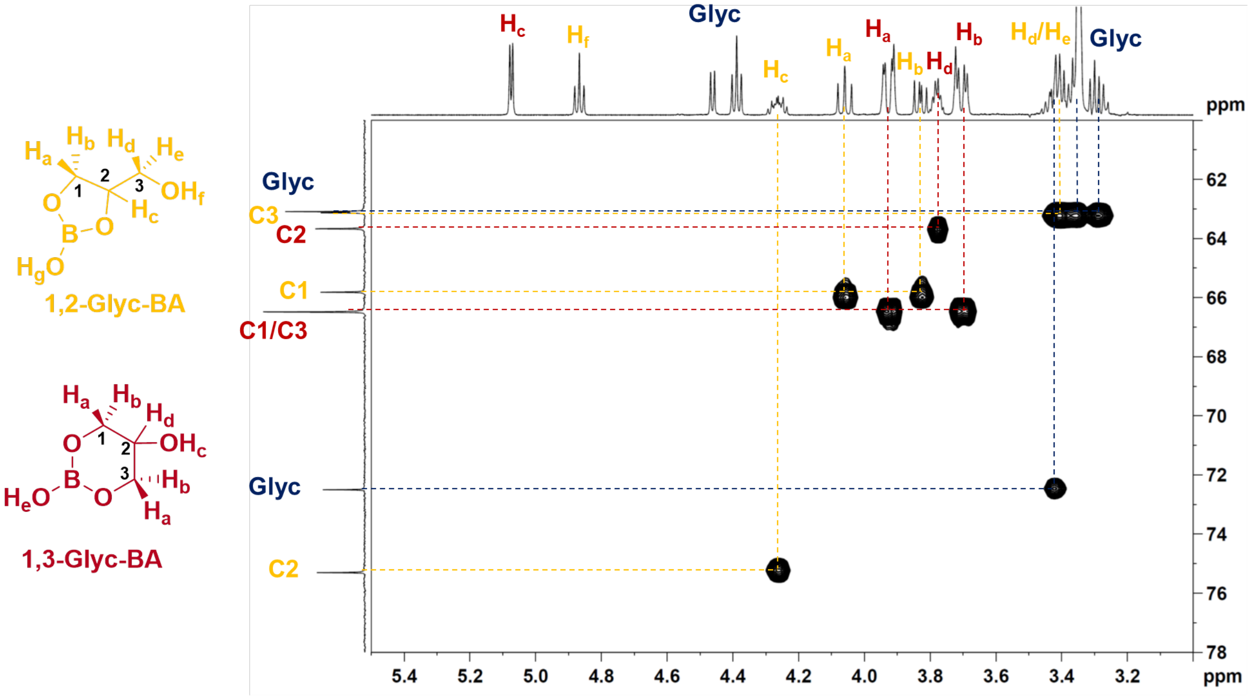

SI-Figure 7. HSQC of 50.0 mM Glyc and 50.0 mM BA in d6-DMSO recorded at 400 MHz (9.4 T).

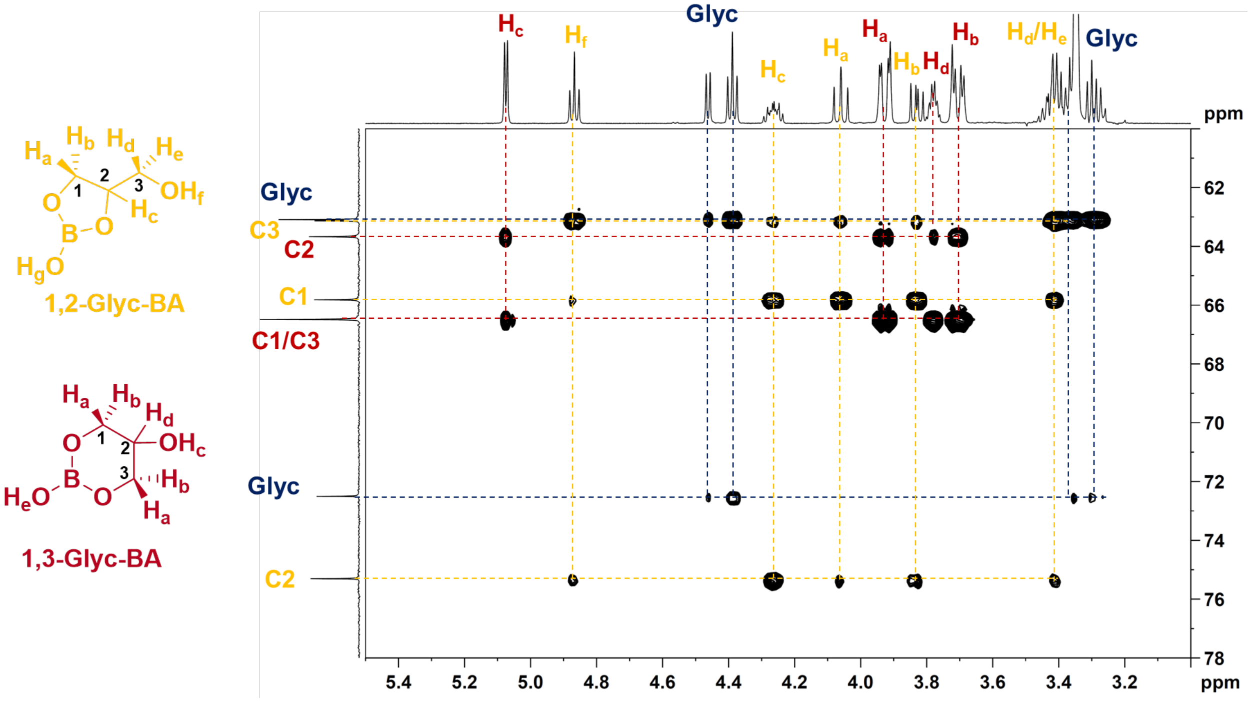

SI-Figure 8. HSQC-TOCSY of 50.0 mM Glyc and 50.0 mM BA in d6-DMSO recorded at 400 MHz (9.4 T).

Isomer	Proton	δ (ppm)	Integration	Multiplicity
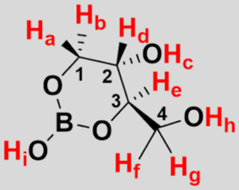 1,3-Ery-BA (◆)	H_a	3.6222	1.0477	dd
	H_b	3.9141	1.0000*	dd
	H_c	5.1011	0.9036	d
	H_d	3.6665	0.9173	m
	H_e	3.7120	0.9878	q
	H_f/H_g	3.4524	1.8358	td
	H_h	4.7503	0.8957	t
	H_i	7.2112	0.8855	s
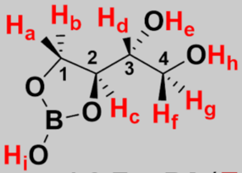 1,2-Ery-BA (■)	H_a/H_b	3.9898	0.4547	dd
	H_c	4.2037	0.2413	q
	H_d	3.4806	0.2189	t
	H_e	4.9485	0.1677	d
	H_f/H_g	3.3550	-	-
	H_h	4.5600	0.1726	t
	H_i	8.0805	0.1634	s

SI-Figure 9. ^{1}H NMR spectral details of 50.0 mM Ery and 100.0 mM BA in d6-DMSO recorded at 400 MHz (9.4 T). *The integration for H_b of the 1,3-Ery-BA complex was 1.0000.

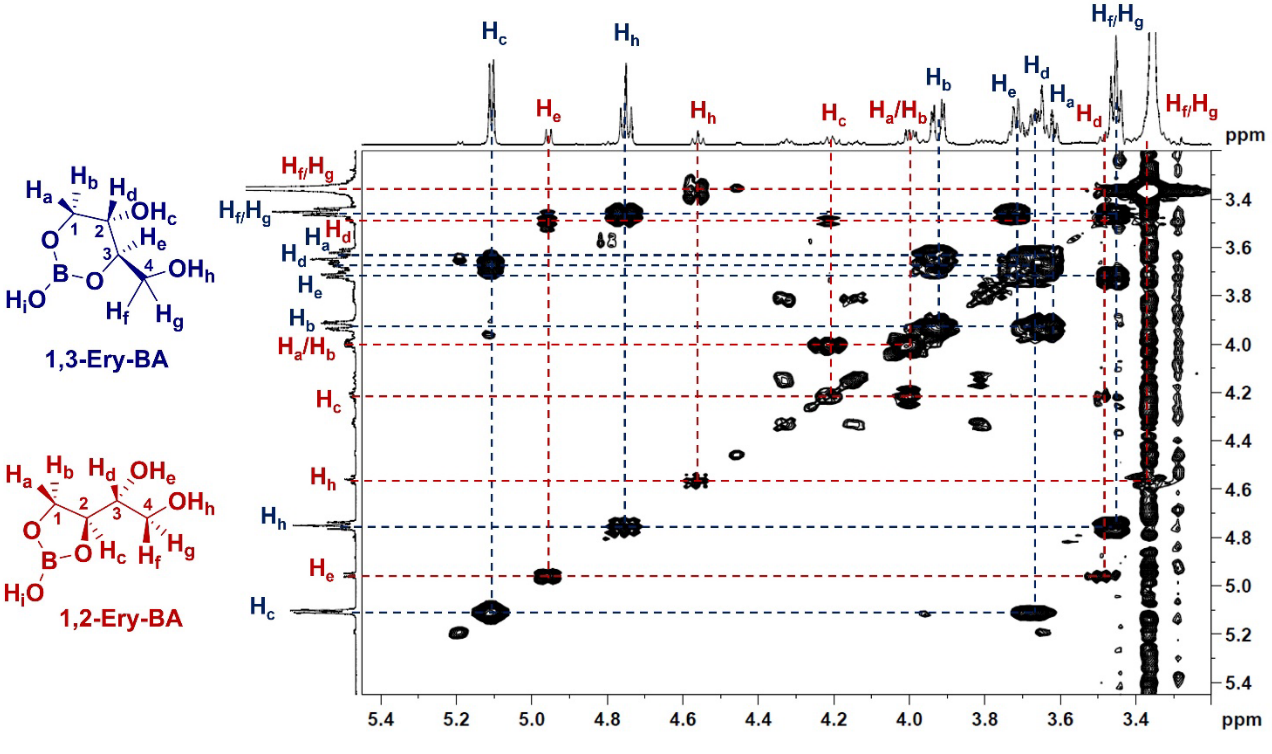

SI-Figure 10. COSY of 50.0 mM Ery and 100.0 mM BA in d6-DMSO recorded at 400 MHz (9.4 T).

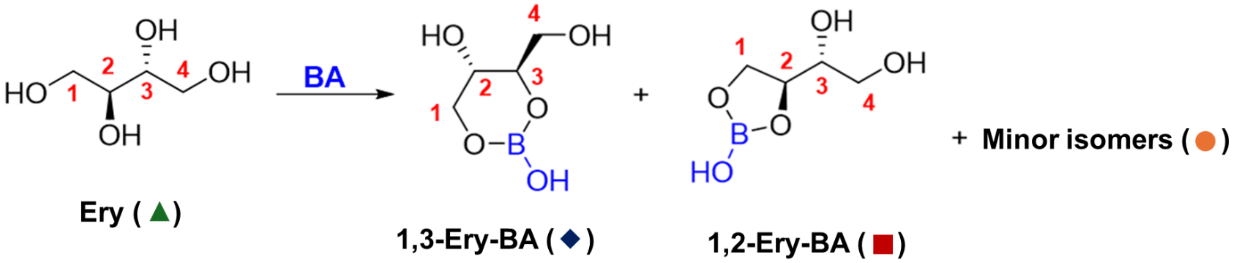

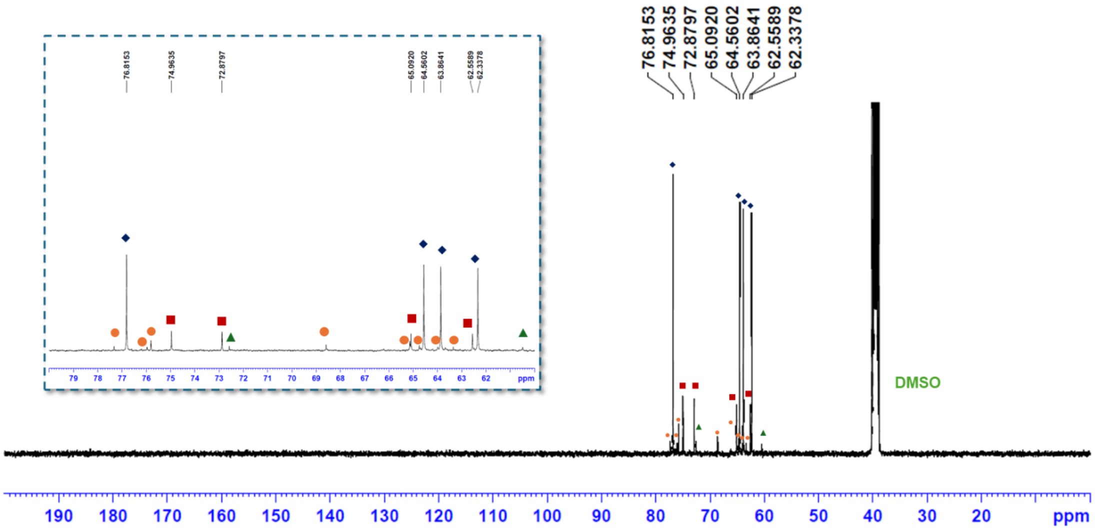

Isomer	Carbon	δ (ppm)
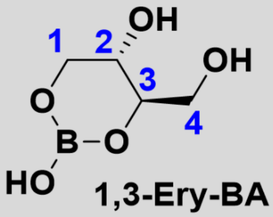 1,3-Ery-BA (◆)	C1	64.5602
	C2	63.8641
	C3	76.8153
	C4	62.3378
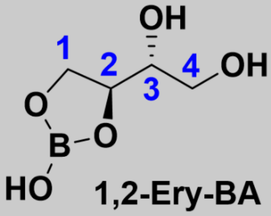 1,2-Ery-BA (■)	C1	65.0920
	C2	74.9635
	C3	72.8797
	C4	62.5589

SI-Figure 11. ^{13}C NMR spectrum and spectral details of 50.0 mM Ery and 100.0 mM BA in d6-DMSO recorded at 400 MHz (9.4 T).

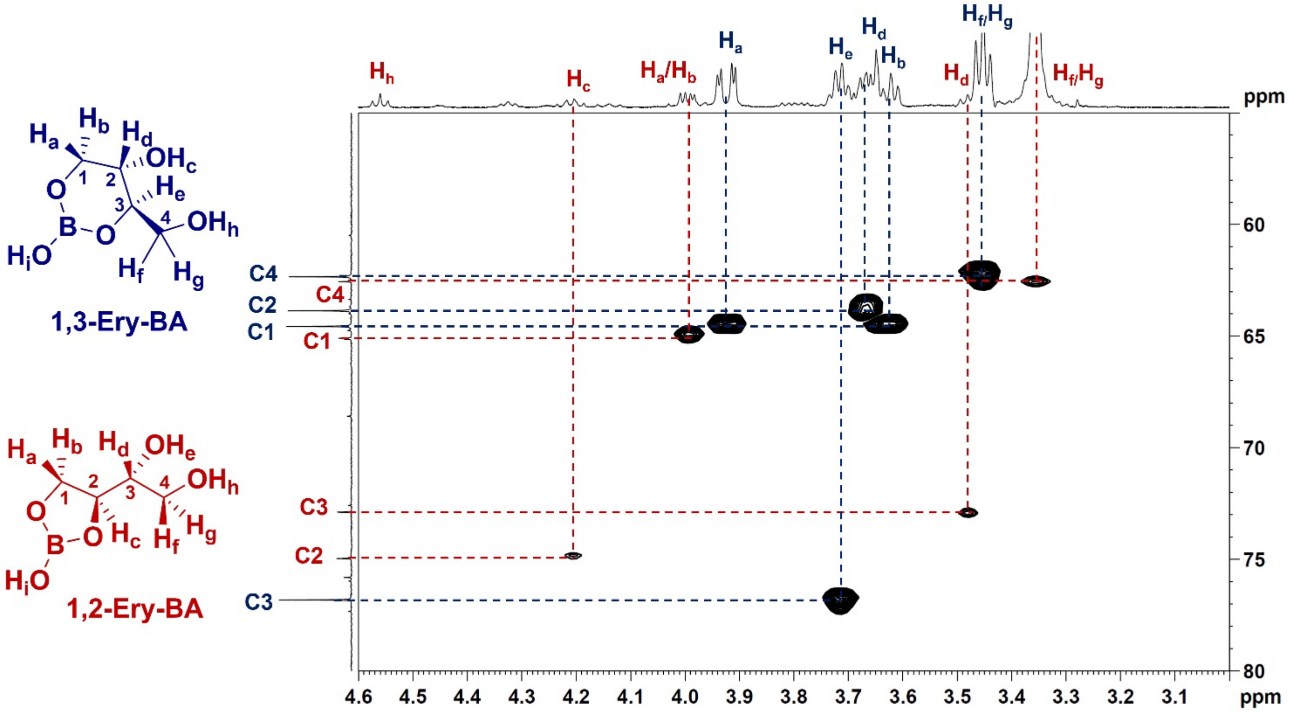

SI-Figure 12. HSQC of 50.0 mM Ery and 100.0 mM BA in d6-DMSO recorded at 400 MHz (9.4 T).

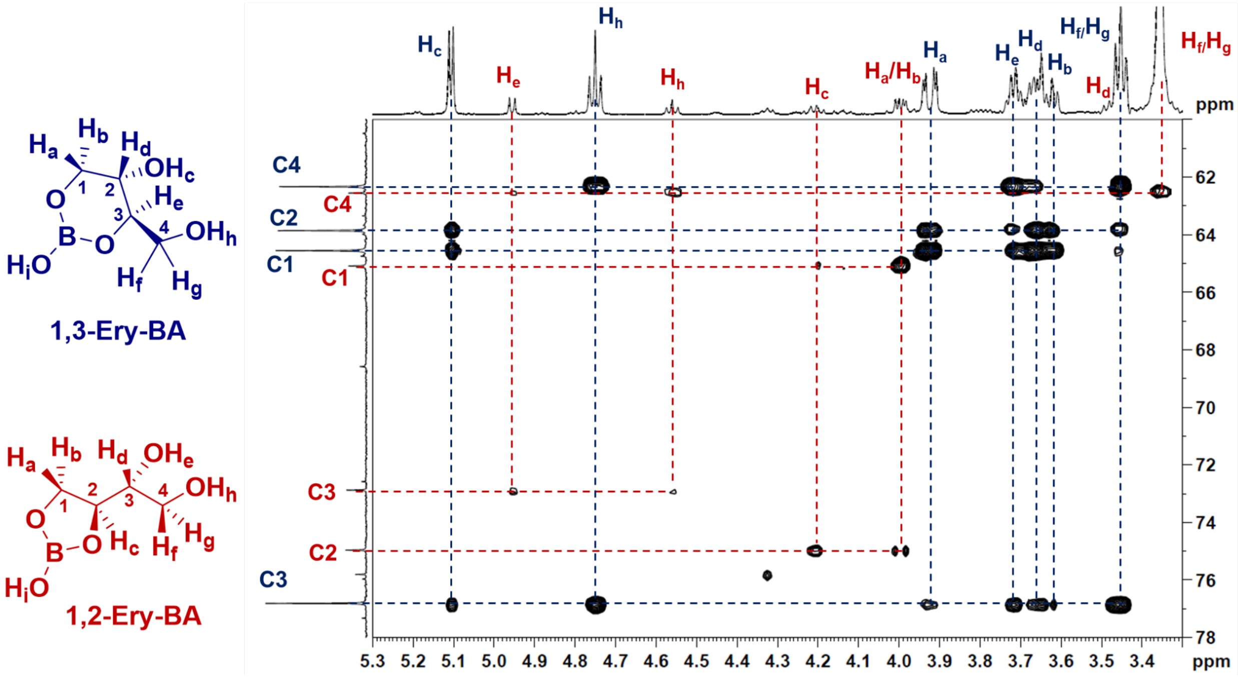

SI-Figure 13. HSQC-TOCSY of 50.0 mM Ery and 100.0 mM BA in d6-DMSO recorded at 400 MHz (9.4 T).

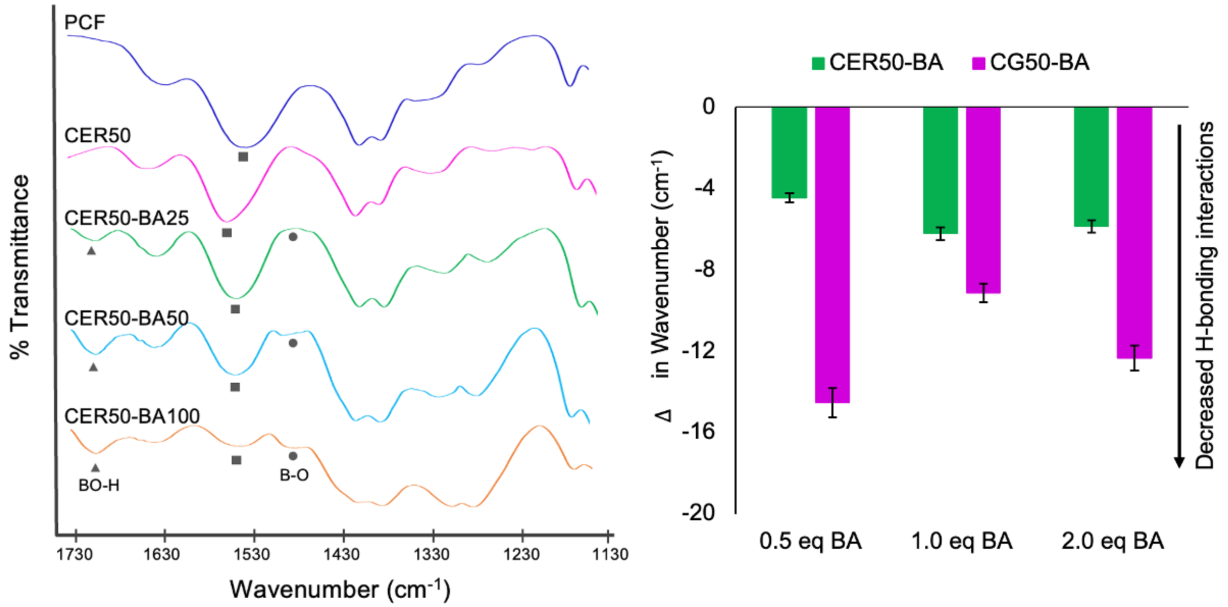

SI-Figure 14. (Left) ATR-FTIR spectra of the amide II region of PCF (blue), CER50 (pink), CER50-BA25 (green), CER50-BA50 (blue), and CER50-BA100 (orange). Triangle and circle annotations highlight frequencies from boric acid and neutral boron complexes, respectively. Squares highlight the OH bend of chitosan. (Right) Plot of change in wavenumber (Final cm-1 - Initial cm-1) of CER50-BA (green) and CG50-BA (fuchsia) with increasing concentration of boric acid (0.5-2.0 eq.).

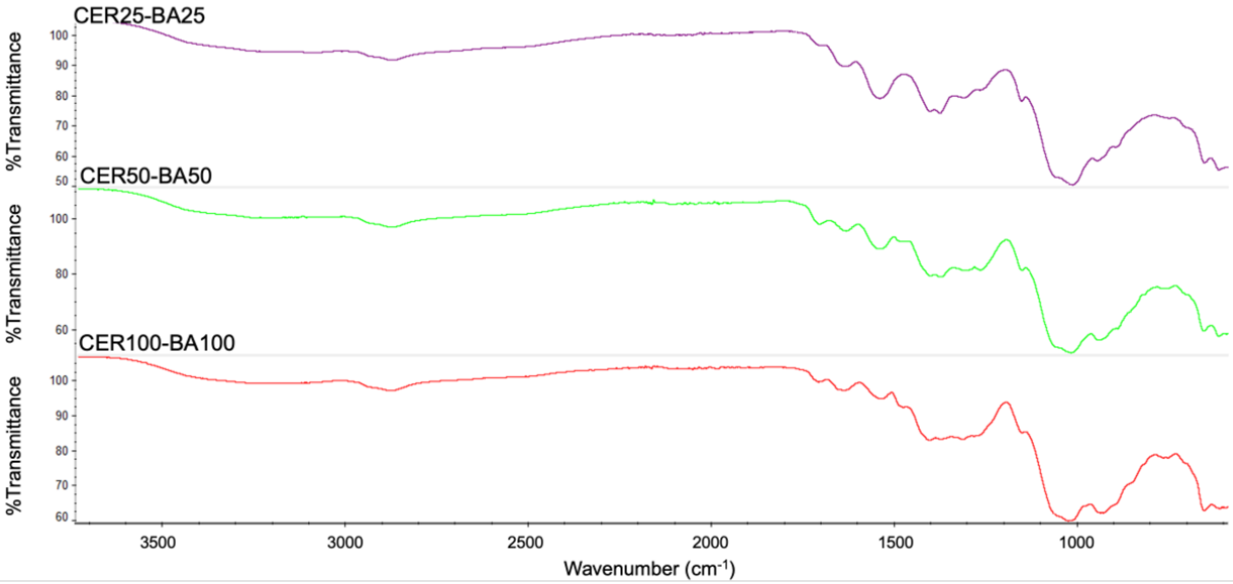

SI-Figure 15A. Full ATR-FTIR spectra of CER25-BA25 (purple), CER50-BA50 (green), and CER100-BA100 (red).

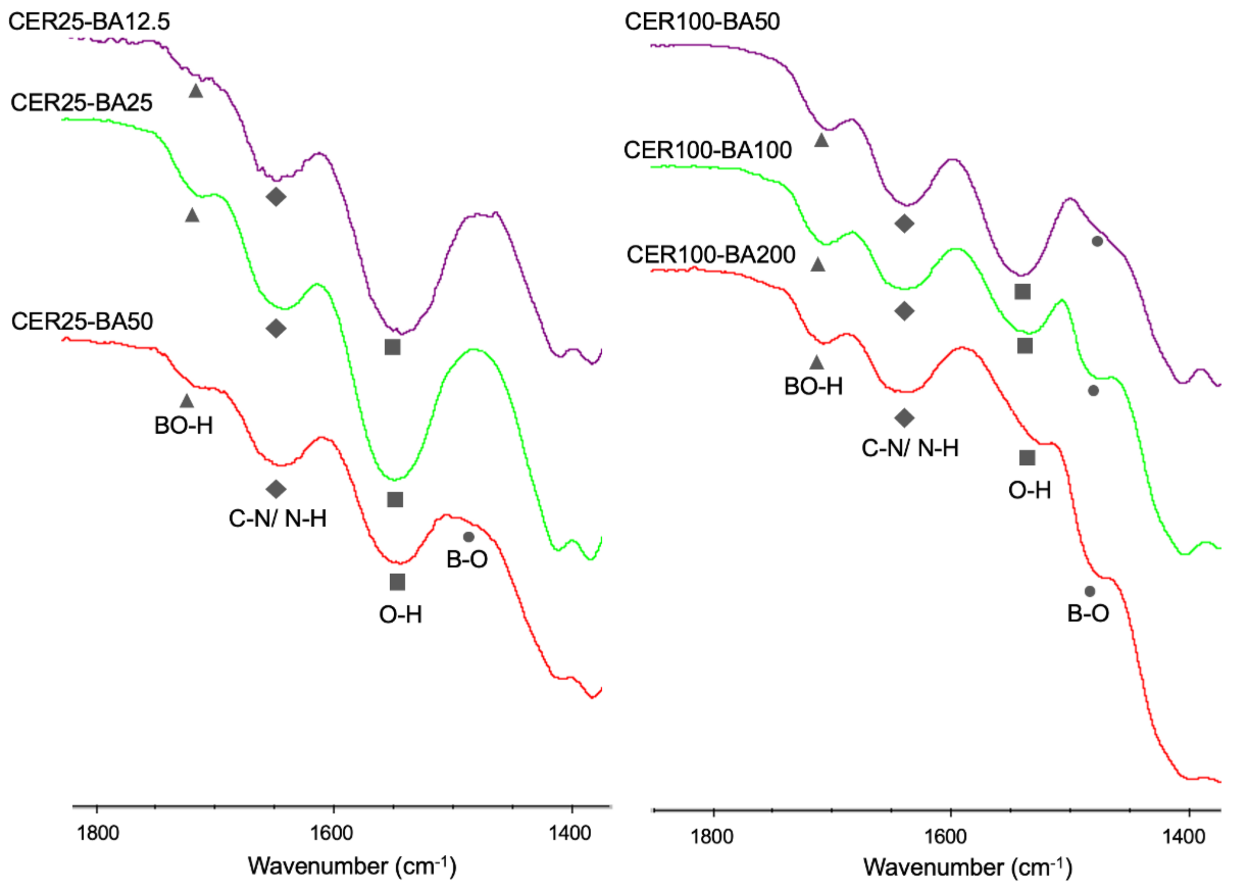

SI-Figure 15B. ATR-FTIR spectra of the amide II region of CER25-BA12.5 (purple), CER25-BA25 (green), CER25-BA50 (red), CER100-BA50 (purple), CER100-BA100 (green), and CER100-BA200 (red). Triangle and circle annotations highlight frequencies from boric acid and neutral boron complexes, respectively. Diamonds and squares highlight the CN/NH combination band and the OH bend of chitosan, respectively. Presence of B-O stretching frequency indicates the precedence of Ery-BA complexes in the films.

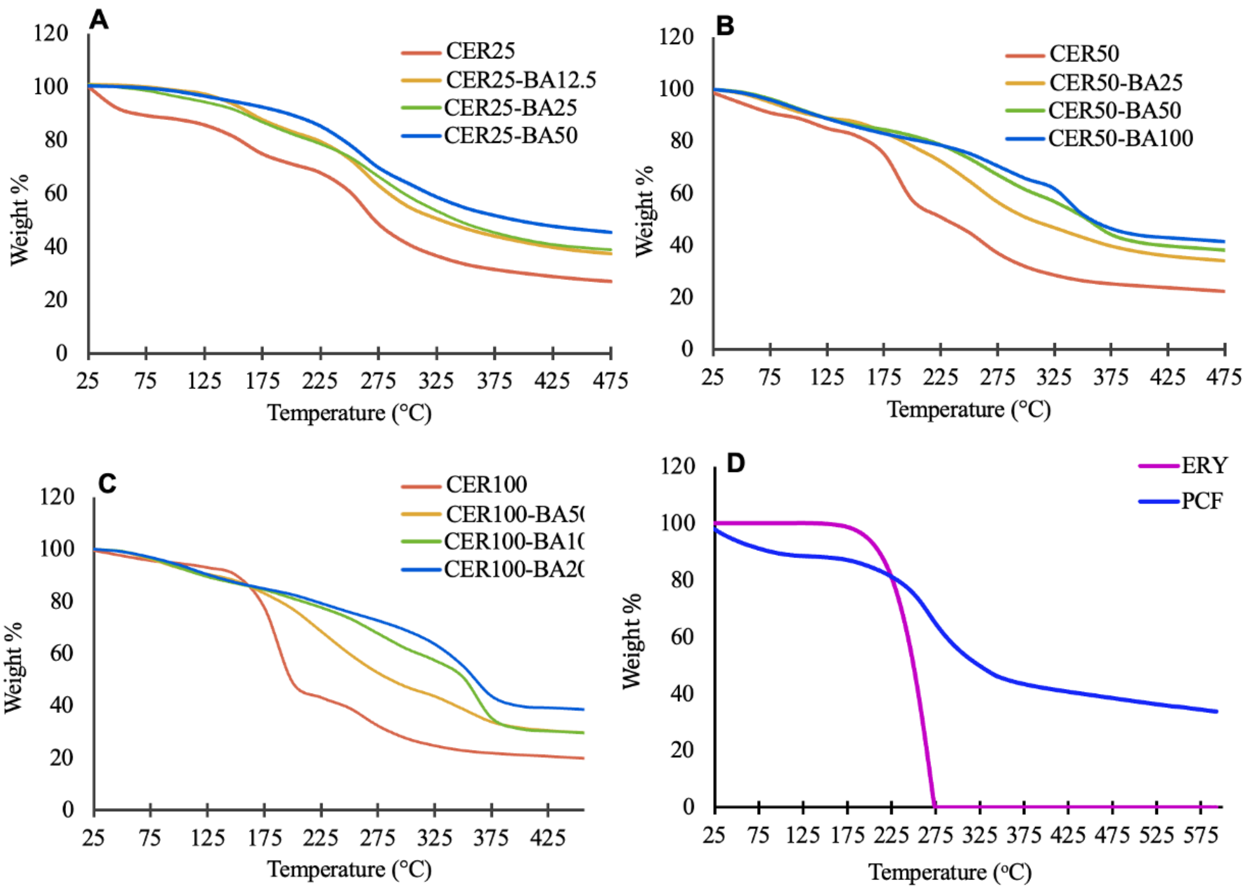

SI-Figure 16. TGA weight loss curves of (A)CER25 and CER25-BA12.5-50 films, (B)CER50 and CER50-BA25-100 films, (C)CER100 and CER100-BA50-200 films, and (D) pure erythritol (ERY) and PCF.

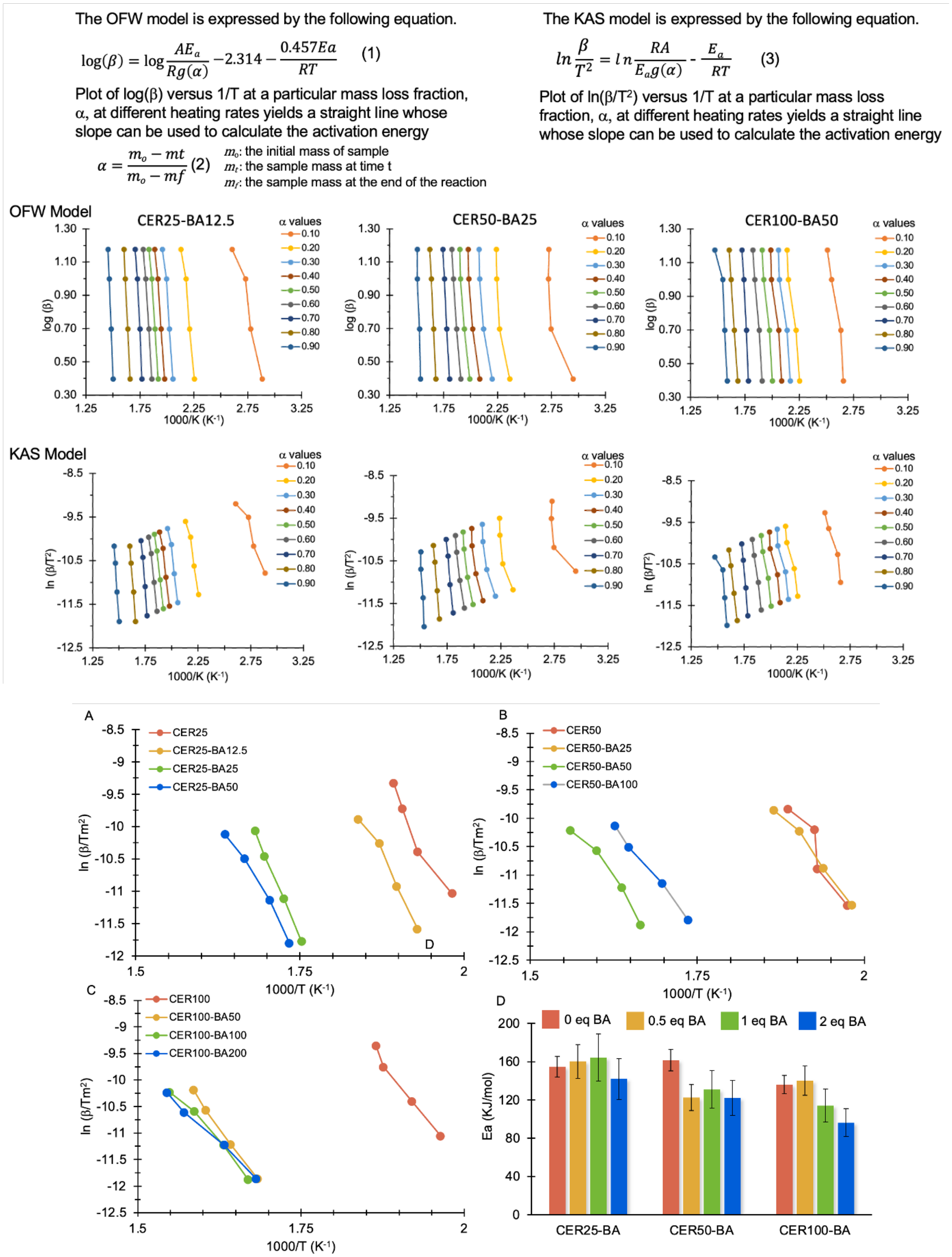

SI-Figure 17. (Top) Equations and plot description for obtaining activation energies (E_a) from OFW and KAS isoconversional models. Sample OFW and KAS plots from CER25-BA12.5, CER50-BA25, and CER100-BA50 with alpha values of 0.1-0.9. (Bottom) KAS plot for chitosan decomposition step of (A) CER25 and CER25-BA12.5-50 (B) CER50 and CER50-BA25-100, and (C) CER100 and CER100-BA5-200 as well as (D) a plot of Ea values for all films with error bars.

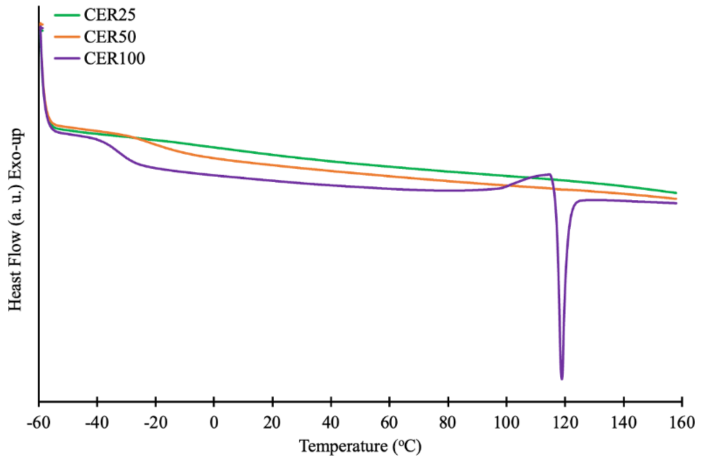

SI-Figure 18. DSC second heating curve of CER25 (green) CER50 orange and CER100 (purple).

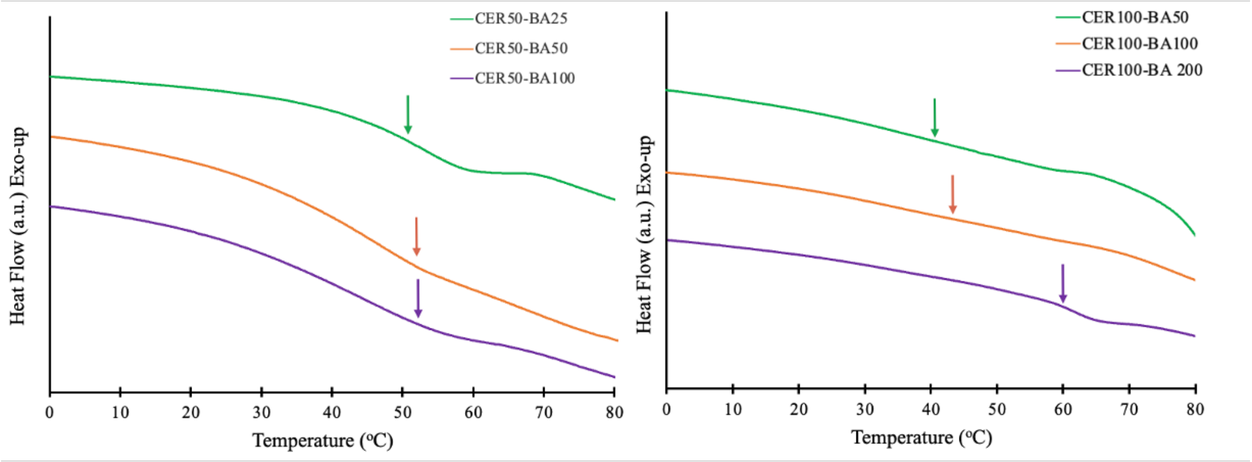

SI-Figure 19. DSC second heating curves of CER50 (left) and CER100 (right) series with increasing BA.

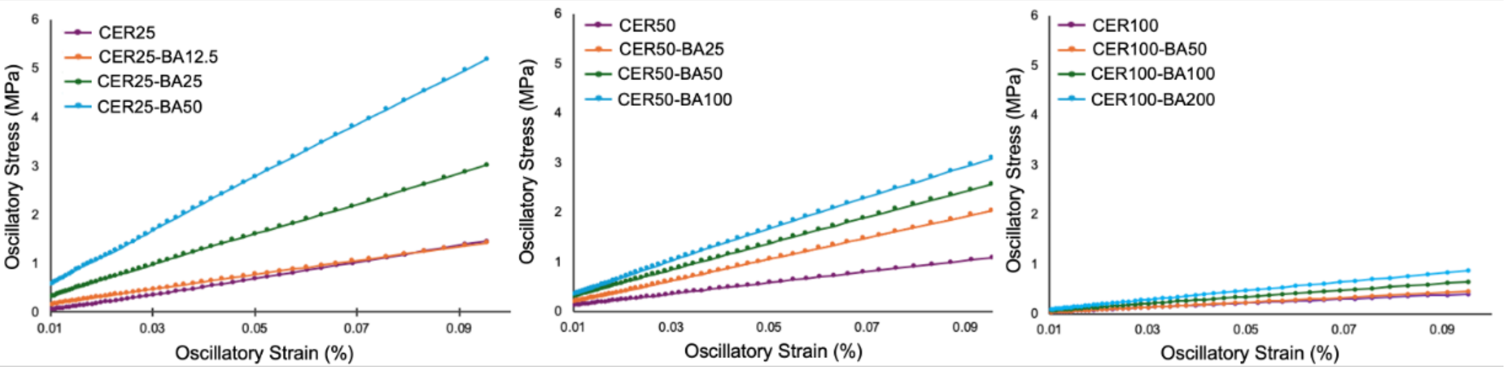

SI-Figure 20. DMA Young's modulus plots of CER25-BA12.5-50 (left), CER50-BA25-100 (middle), and CER100-BA50-200 (right). CER25-BA films are the stiffest with the highest Young's modulus values while CER100-BA materials show a significant decrease in Young's modulus indicating an increase in flexibility.

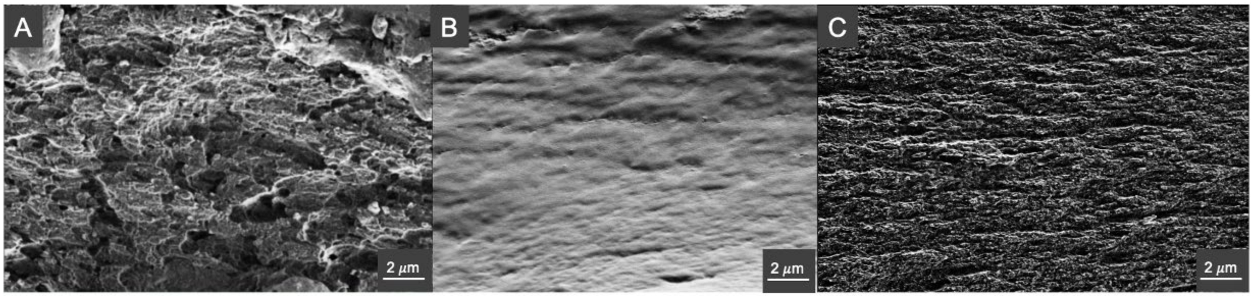

SI-Figure 21. SEM cross-sectional images of (A)PCF, (B)CG50, and (C) CG50-BA50. Images combined from previous publications.[1,2]

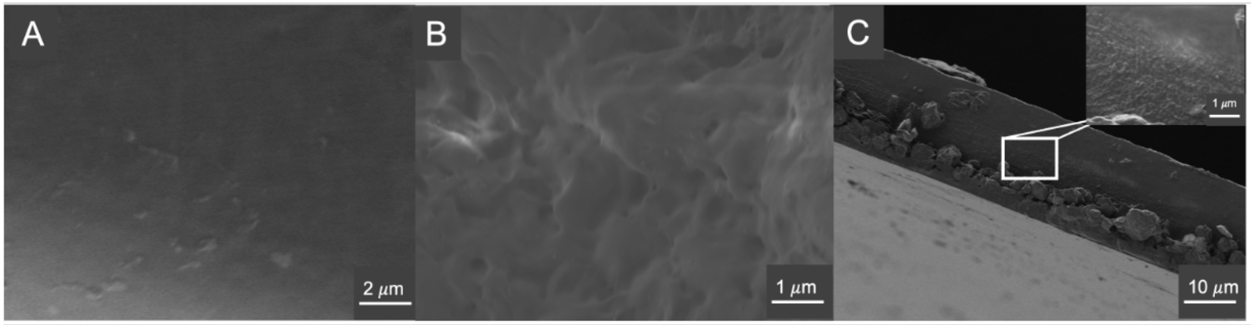

SI-Figure 22. SEM cross-sectional images of (A) CER25-BA12.5, (B)CER25-BA25, and (C) CER25-BA50. Image A is shown at 5KX, image B is shown at 10KX and image C is shown at 1KX with an insert of a 10KX image. Images at different magnifications to ensure the best resolution while highlighting the increase in roughness of the material with increasing BA.

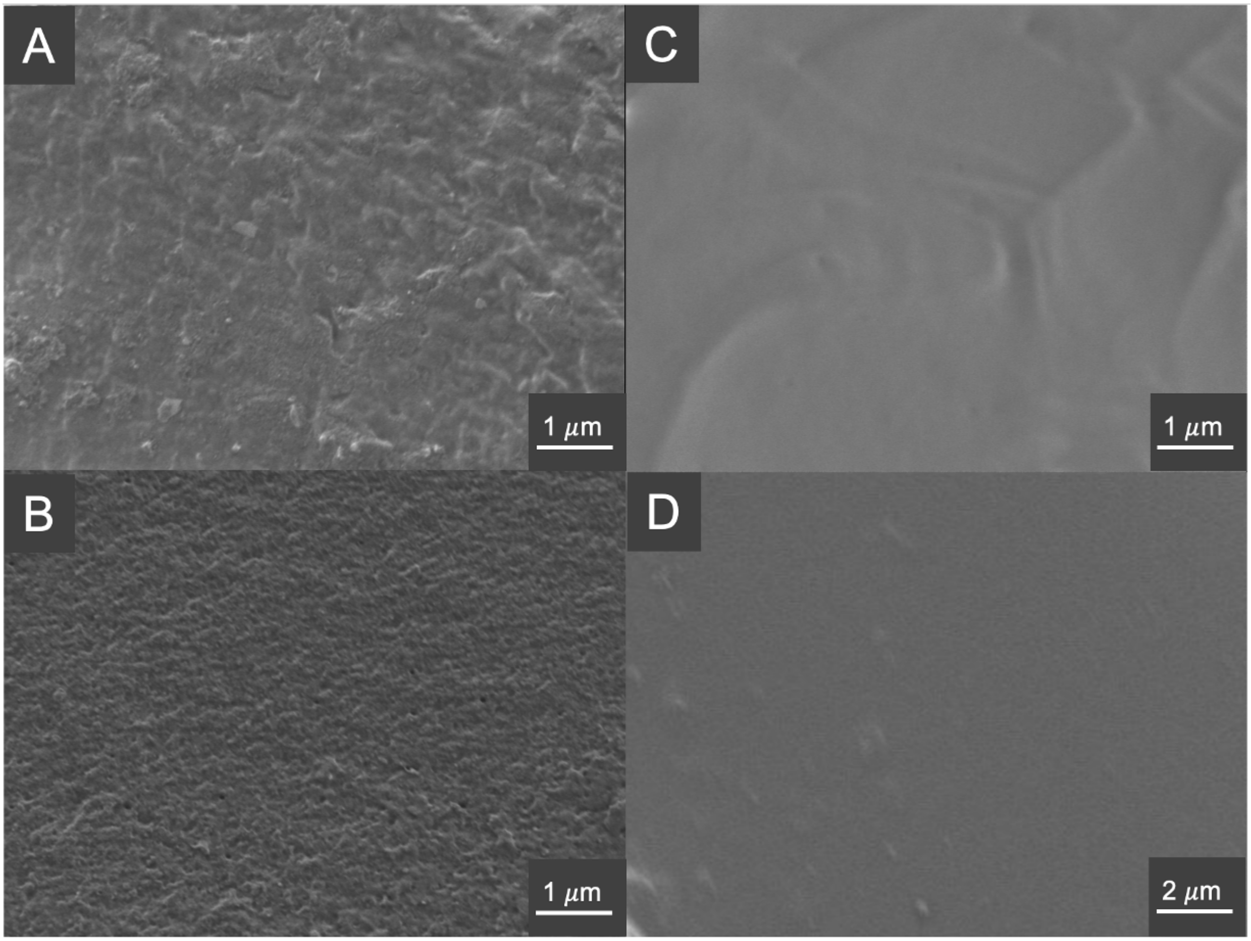

SI-Figure 23. Cross-sectional SEM images of (A) CER50-BA25, (B) CER50-BA50, (C) CER100-BA50, and (D) CER100-BA200. Images A-C are shown at 10KX magnification while D is shown at 5KX magnification. The smoothness of CER100-BA200 (D) films are such that increased magnification resulted in poorly resolved images at increased magnification.

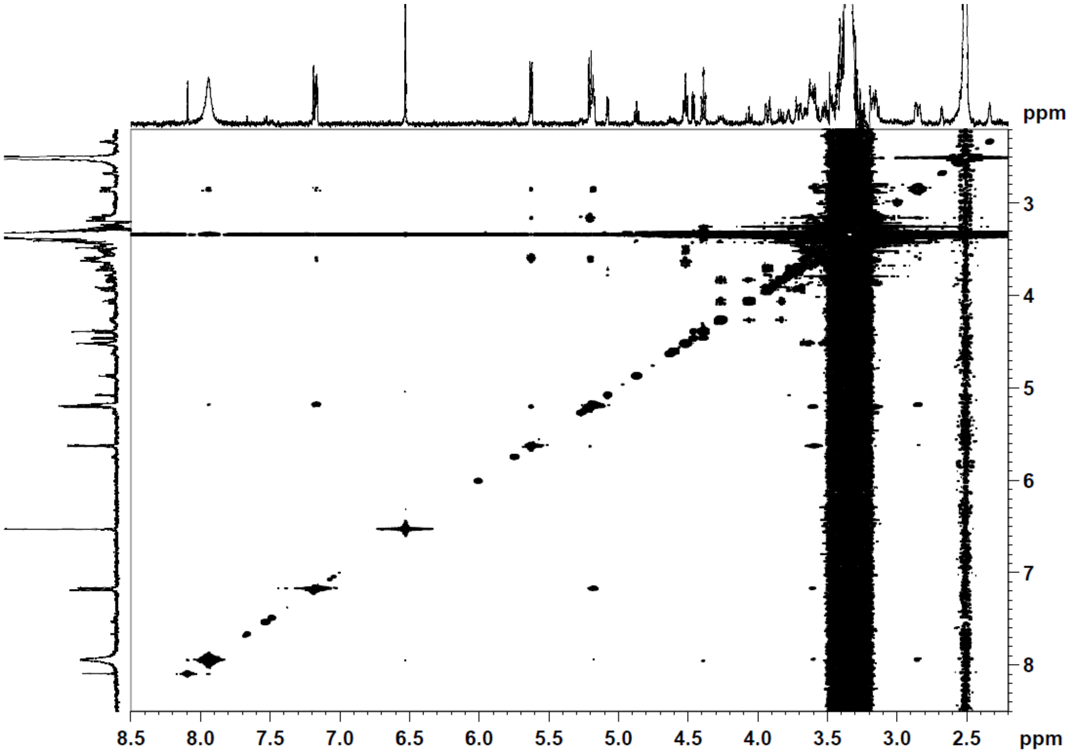

SI-Figure 24. Full NOESY spectrum of 3.0 mM GlcN, 3.0 mM Glyc, and 3.0 mM BA in d6-DMSO recorded at 400 MHz (9.4 T).

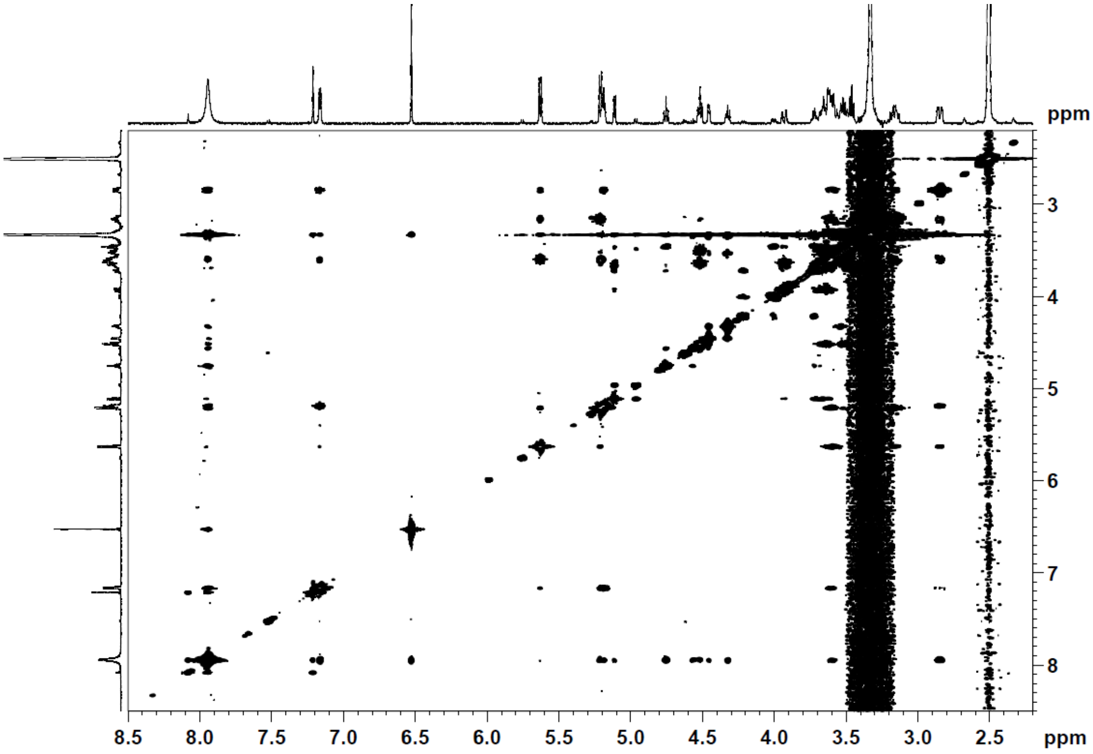

SI-Figure. 25. Full NOESY spectrum of 3.0 mM GlcN, 3.0 mM Ery, and 3.0 mM BA in d6-DMSO recorded at 400 MHz (9.4 T).

SI-References

[1] Scott, H.R.; Pearson, C. J.; Ealley, L. C.; Boardman, B. M.; Peters, G. M. *International J. of Biol. Macromolecules* **2024**, *258*, 129304.

[2] Mars, S. W.; Davidson, B. L.; Moore, K. H.; Boardman, B. M.; Peters, G. M. *ACS Omega*, **2024**, *9*, 41687-41695.
